# Freshwater and anadromous fishing in Ice Age Beringia

**DOI:** 10.1126/sciadv.adg6802

**Published:** 2023-06-02

**Authors:** Ben A. Potter, Carrin M. Halffman, Holly J. McKinney, Joshua D. Reuther, Bruce P. Finney, François B. Lanoë, J. Andrés López, Charles E. Holmes, Erica Palmer, Marie Capps, Brian M. Kemp

**Affiliations:** ^1^ Department of Anthropology, University of Alaska Fairbanks, Fairbanks, AK, USA.; ^2^ Archaeology Department, University of Alaska Museum of the North, Fairbanks, AK, USA.; ^3^ Department of Biological Sciences and Geosciences, Idaho State University, Pocatello, ID, USA.; ^4^ Bureau of Applied Research in Anthropology, University of Arizona, Tucson AZ, USA.; ^5^ College of Fisheries and Ocean Sciences, University of Alaska Fairbanks, Fairbanks, AK, USA.; ^6^ Department of Fishes and Marine Invertebrates, University of Alaska Museum of the North, Fairbanks, AK, USA.; ^7^ Laboratories of Molecular Anthropology and Microbiome Research, Norman, OK, USA.; ^8^ Department of Anthropology, University of Oklahoma, Norman, OK, USA.

## Abstract

While freshwater and anadromous fish have been critical economic resources for late prehistoric and modern Native Americans, the origin and development of fishing is not well understood. We document the earliest known human use of freshwater and anadromous fish in North America by 13,000 and 11,800 years ago, respectively, from primary anthropogenic contexts in central Alaska (eastern Beringia). Fish use appears conditioned by broad climatic factors, as all occurrences but one are within the Younger Dryas chronozone. Earlier Bølling-Allerød and later early Holocene components, while exhibiting similar organic preservation, did not yield evidence of fishing, suggesting that this was a response to changing environmental factors, perhaps reductions in higher ranked resources such as large terrestrial mammals. Late Pleistocene and recent Indigenous peoples harvested similar fish taxa in the region (salmon, burbot, whitefish, and pike). We characterize late Pleistocene fishing in interior Beringia as an important element of broad-spectrum foraging rather than the intensive communal fishing and storage common among recent peoples.

## Teaser

Ice Age Beringians in central Alaska harvested freshwater fish by around 13,000 years ago and salmon by around 11,800 years ago.

## INTRODUCTION

Freshwater and anadromous fish have long been important staple resources for Native Americans; however, little is known about the origin and development of the earliest inland fishing in North America. Early lifeways [late Pleistocene and early Holocene, ca. 14,000 to 7000 calendar years before present (cal yr B.P.)] tend to be dichotomized as megafaunal specialization or broad-spectrum foraging, but fish are rarely mentioned [e.g., (*1*, *2*)]. While this may reflect subsistence practices, the archaeological record may be unrepresentative due to taphonomic issues such as poor preservation or recovery rate leading to the absence of tiny, fragile fish remains from zooarchaeological assemblages (*3*). While freshwater fish remains have been reported from a few late Pleistocene–aged inland sites in North American south of the continental glaciers (*4*–*7*), these occur either in caves or along water margins where distinguishing between natural and cultural accumulations of fish is difficult or where fish has been misidentified (*8*–*11*).

In contrast, Beringia is an area where early Paleoindian fishing adaptations could be observed, and it occupies a key geographic position in current peopling of the Americas models. Beringia was mostly ice-free during the last glacial period and was a major refugium for a variety of fish taxa (*12*–*14*), and open-air loess sites with excellent organic preservation have the potential to preserve delicate fish remains. Very little is known about early fishing in Beringia, and reports are generally limited to the presence/absence of fish with no further taxonomic specificity (*15*, *16*). One exception is our work at Upward Sun River, where we identified early use of chum salmon (*Oncorhynchus keta*) through zooarchaeological, genetic, and isotopic analyses (*17*–*19*). A few other sites contain fish but lack chronological controls and/or demonstration of anthropogenic origin of the assemblages (*20*–*22*). Understanding early fishing in Beringia can address two current problem areas: economic adaptations relating to the peopling of the Americas and economic change through time in the region as humans responded to climatic oscillations during the Pleistocene-Holocene transition.

**Fig. 1.**
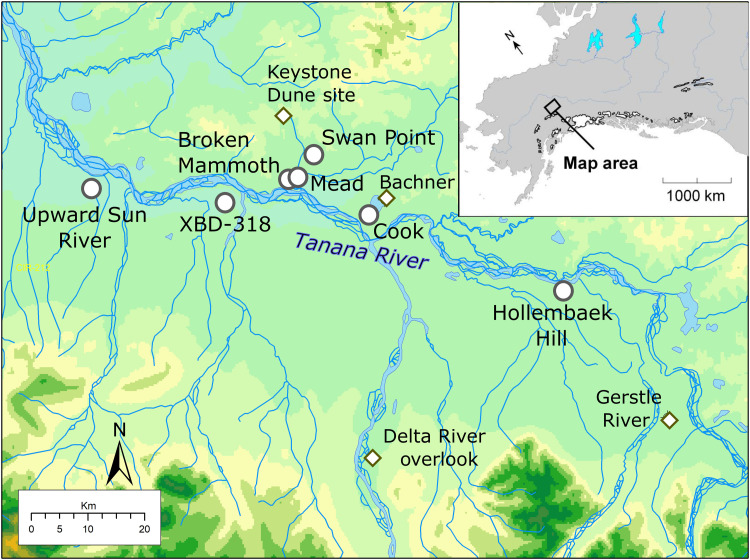
Site locations in Eastern Beringia, middle Tanana River valley. Fish-bearing sites are shown as circles, and other sites mentioned in the text are shown as diamonds.

Here, we present zooarchaeological and biomolecular analyses of fish remains from several archaeological sites in interior Alaska (eastern Beringia). We conducted a comprehensive review of all sites 7000 years or older in interior Alaska (Tanana, Kuskokwim, Susitna, and Copper River basins) for reports of fish. This review yielded 10 archaeological components, all from the middle Tanana basin, eight of which had fauna available for study (Fig. 1 and Table 1). The Tanana basin encompasses the densest concentration of late Pleistocene sites in the Americas, with 38 components dating >11,500 cal B.P. and 18 dating to Clovis or earlier periods (>13,000 cal B.P.) (*23*–*25*). This region can be considered representative of interior Beringian paleoenvironments, heavily used by early peoples, as 79% of late Pleistocene sites in Beringia are located adjacent to large interior rivers, while there are no known Beringian coastal sites (*26*). With numerous expansive surveys and large-scale excavations of multicomponent sites with good organic preservation, interior Alaska provides an exceptional window into broader Beringian human adaptations. The major cultural manifestations are widespread in eastern Beringia, with connections to western Beringia (*27*, *28*).

**Table 1.** Evidence for late Pleistocene to early Holocene fishing in the Tanana Valley, Alaska. See Supplementary Materials and table S2 for details of radiocarbon dating. NSP (number of specimens) includes all specimens that could be assigned to a fish class without the aid of magnification; NISP (number of identified specimens) includes all specimens that could be identified to taxonomic order or lower, including “likely” identification. Unidentified specimens are fragments that could be attributed to fish but not further specified.

Site	Age (cal B.P.)	Season	Burbot	Whitefish	Pike	Salmon	Unidentified	NSP	NISP
Mead C2b	13,110–12,790	?	5	–	–	–	11	16	5
Swan Point CZ3b	12,790–12,520	?	1	–	–	–	–	1	1
Cook CZ3	12,530–12,050	?	–	–	2	–	–	2	2
Broken Mammoth CZ3*	12,390–11,770	Fall?	–	–	–	–	Present	Present	–
Mead C3a (CZ3b2)	12,200–11,820	Spring	356	23	–	–	327	706	379
Upward Sun River C2^†^	11,990–11,510	Summer/fall	–	–	–	Present	–	–	–
XBD-318	~12,000–11,300	Summer/fall	–	–	–	1	–	1	1
Swan Point CZ3a	11,890–11,430	?	2	–	–	–	–	2	2
Upward Sun River C3	11,610–11,280	Summer	–	11	–	200	145	356	211
Hollembaek’s Hill	8,800–7,890	Multiple	1	13	1	11	–	26	26
**Total**			**365**	**47**	**3**	**212**	**483**	**1110**	**627**

*Broken Mammoth fish were reported but not firmly identified to taxon (*16*) and were unavailable for study.

†Presence of salmon based on chemical profiling of hearth sediments; no fish bones were recovered from this component (*19*).

Zooarchaeological analyses included taxonomic and body part identifications, as well as bone modification and taphonomic analyses, to characterize the fish assemblages, evaluate factors relating to preservation and processing by humans, and ultimately understand early human fishing behaviors. We complemented the zooarchaeological analyses with biomolecular analyses (DNA and carbon and nitrogen stable isotopes) on a subset of specimens selected based on preservation and mass. Biomolecular analyses also incorporated salmon specimens from Lime Hills Cave, a mixed paleontological/cultural assemblage dating to the terminal Pleistocene/early Holocene (*20*, *29*) to identify the salmon species available in the adjacent Kuskokwim basin at this early time period, and we do not include this site among those with clearly anthropogenic fish assemblages. Temporal patterns in human use of freshwater and anadromous fish are examined in the context of regional climate and vegetation change and in relation to well-documented fishing data from recent local Alaska Native communities. These analyses allow for a hypothetical reconstruction of Pleistocene fishing adaptations, including targeted species, seasonality, technology, and mobility strategy.

## RESULTS

**Fig. 2.**
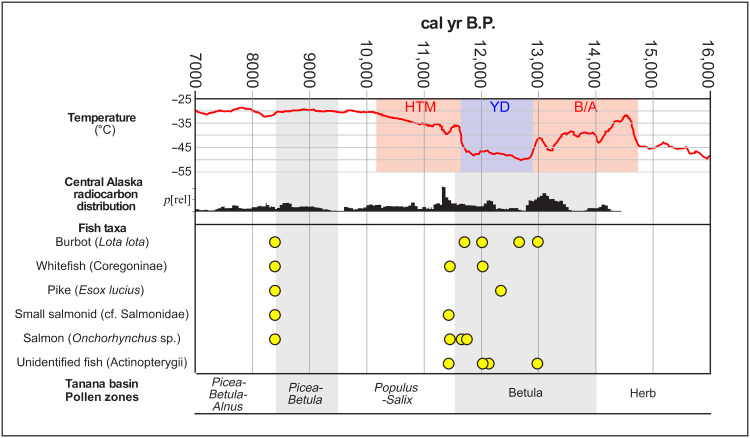
Fish taxa, human population, climate, and vegetation proxies for central Alaska. Yellow dots represent midpoints of two sigma age ranges for fish occurrences within archaeological sites in this study (see the Supplementary Materials and table S2). Temperature from (*94*). Central Alaska radiocarbon distribution is a summed ^14^C distribution of dated components for the region (*95*). Pollen zone data from (*61*). HTM, Holocene thermal maximum; YD, Younger Dryas); B/A, Bølling-Allerød.

The eight components with identified fish remains are found at six open-air sites within stratified loess deposits on raised landforms (e.g., bedrock knolls, and sand dunes) (Fig. 2 and see the Supplementary Materials and table S1). All but one of the components date to the Younger Dryas chronozone of the last glacial (~12,900 to 11,650 cal yr B.P.) (table S2). These sites were excavated by B.A.P. (Upward Sun River, Mead, and XBD-318), C.E.H. (Swan Point and Broken Mammoth), and F.B.L. and J.D.R. (Cook and Hollembaek’s Hill) using similar recovery methods. All identified fish taxa from these late Pleistocene–early Holocene assemblages are present in the Tanana River today (see the Supplementary Materials). A total of 1110 fish specimens were identified, all assigned to the class Actinopterygii (ray-finned fishes) (Table 1 and dataset S1). Of these, 627 (56%) could be identified to a lower taxonomic level. Identified taxa include anadromous *Oncorhynchus* spp. (salmon, 34% of the overall identified assemblages) and three freshwater taxa: *Lota lota* (burbot; 58%), subfamily Coregoninae [whitefish, 7%, including seven (1%) identifiable to *Coregonus pidschian* or humpback whitefish], and *Esox lucius* (northern pike; <1%). All of these taxa are used for subsistence today in the Tanana (see Discussion and table S3), and most are widespread in northern North America, beyond Beringia. Notably, we did not identify other modern subsistence fish: *Thymallus arcticus* (grayling), *Salvelinus* spp. (char), or *Catostomus catostomus* (longnose sucker).

All identified fish before 11,800 cal yr B.P. are freshwater fish, mostly burbot, representing the earliest evidence of fishing in Beringia, and occur at Mead C2b (~12,950 cal yr B.P.) and Swan Point CZ3b (~12,600 cal yr B.P.). Pike, whitefish, and salmon appear by 12,300, 12,000, and 11,800 cal yr B.P. respectively. Fish abundance is uneven across the samples, with Mead C3a (~12,000 cal yr B.P.) containing 379 number of identified specimens (NISP), mostly burbot, and Upward Sun River C3 (~11,000 cal yr B.P.) containing 211 NISP, mostly salmon. All other sites contain 25 or fewer fish NISP. Fish richness, diversity, and evenness also vary across the samples (table S4). Only Mead, Upward Sun River, and Hollembaek provide adequate samples to consider diversity and evenness; the other sites are composed of just a single taxon and sample sizes less than 5. Diversity (*H* = 0.205 to 0.229) and evenness (*E* = 0.330 to 0.295) are very low for Mead and Upward Sun River, respectively, while Hollembaek’s Hill is more diverse and more even (*H* = 1.05, *E* = 0.757).

Most fish specimens are bone elements (*n* = 1078, 97%), although 24 teeth, 1 tooth in the bone, and 7 otoliths were also identified. No fish scales were recorded. Of the specimens identified to element (*n* = 744), most (84%) are vertebrae, with 15% cranial, 1% pectoral/pelvic, and no tail parts (table S5). Body part representation for bone elements is very similar between the burbot assemblage at Mead C3a and the salmon assemblage at Upward Sun River C3 in being predominantly vertebral (77 and 81%, respectively), with some cranial (22 and 19%, respectively) and ~1% pectoral/pelvic (table S6). Identifiability indices (proxy measures of the degree of bone fragmentation) are also similar between Mead C3a and Upward Sun River C3, including number of specimens (NSP)/NISP (1.9 and 1.7, respectively) and the identification rate (*30*) (53.7 and 59.3%, respectively). These similarities in body part representation and fragmentation suggest that similar processing occurred at both sites; however, the Upward Sun River C3 assemblage is mostly burned (93%), while only 1% of the Mead C3a assemblage is burned, suggesting differential disposal strategies.

Ancient DNA methods were used to recover DNA from 19 specimens for genetic species identification using a “universal” barcode located in the mitochondrial 12*S* ribosomal RNA gene. Of these, 11 (58%) were identified to the species level, with 10 matching the morphological identifications (91% success rate) (see the Supplementary Materials and table S7). These 10 identifications include burbot (*L. lota*) at Mead C3a and Swan Point CZ3b and chum salmon (*O. keta*) from Upward Sun River C3, XBD-318, and the mixed paleontological/cultural Lime Hills Cave site (Genbank accessions OP547324 to OP547329). These results indicate that all sampled taxa align with the genetics of modern species.

We selected 28 specimens for stable isotope analyses, and bone collagen was successfully extracted from 14 specimens (50%), including seven salmon, two burbot, and five whitefish. The relatively high δ^13^C and δ^15^N values of the salmon (all identified as chum salmon, *O. keta*, through DNA analysis) demonstrate feeding in a marine environment (tables S7 and S8). In contrast, the more negative δ^13^C values of the burbot and whitefish indicate a freshwater ecology for these fishes (table S8). The freshwater fishes also show relatively high δ^15^N values, reflecting their high trophic positions (i.e., predators), and are consistent with isotopic studies of modern samples (*19*, *31*). Both stable isotope and DNA analyses were largely unsuccessful on specimens from one assemblage: Mead C3a. Because Mead C3a small mammals and birds presented no issues with biomolecular analyses (*18*), the failure of adequate preservation of the fish may indicate differential taphonomic factors (including fish processing and possibly boiling) that led to biomolecular degradation.

The combined zooarchaeological and biomolecular analyses enable us to estimate seasonality for five components. Anadromous salmon at Upward Sun River C2 and C3, Hollembaek’s Hill, and XBD-318 suggest occupations in the late summer/early fall, while other data at Upward Sun River C3 indicate a more precise estimate of late July/early August (*18*, *32*). Broken Mammoth CZ3 seasonality is estimated to be fall based on dental annuli from large mammals (*23*). Mead C3a occupation is estimated to be spring based on the presence of medullary bone in birds (*33*). Hollembaek’s early Holocene component suggests occupation(s) in both spring (bird eggshell) and late summer/early fall (anadromous salmon).

## DISCUSSION

Our results demonstrate human use of freshwater fish by at least 13,000 cal yr B.P. and anadromous salmon by at least 11,800 cal yr B.P. Multiple freshwater species were exploited during the Younger Dryas period, including burbot, whitefish, and pike. We have increased the number of sites that document early salmon exploitation (*n* = 4), suggesting recurrent salmon use from ~11,800 to 9000 cal yr B.P.. All archaeological fish assemblages reported here are anthropogenic, deriving from well-stratified loess deposits associated with lithics and cultural features (e.g., hearths) and are in primary context (see the Supplementary Materials), and there is no evidence of natural accumulation (e.g., at the largest fish assemblages, Upward Sun River and Mead, burned fish remains were found in direct association with hearths). All fish-bearing sites here are on positive geomorphic landforms (bluffs and terraces), not on river/lake shorelines where natural fish predators occur (e.g., eagles and minks) (*34*). Human fish exploitation was very limited temporally, to the Younger Dryas stadial (reflecting colder and drier conditions), with a notable absence of fish remains during the Bølling-Allerød interstadial and near-absence in the early Holocene (both reflecting warmer and wetter conditions). Several of us have excavated 33 early components (>7000 cal yr B.P.) with excellent preservation in the middle Tanana basin, with 13 dating to the Younger Dryas (Swan Point, Broken Mammoth, Mead, Upward Sun River, Delta River Overlook, Gerstle River, Hollembaek’s Hill, Cook Site, and XBD-318) (*15*, *26*, *32*, *35*–*41*). Fish-bearing components comprise 30% of all occupations earlier than 7000 cal yr B.P., and 69% of occupations during the Younger Dryas (*23*, *32*, *42*–*47*), suggesting that fishing was a common economic strategy in this early period.

**Fig. 3.**
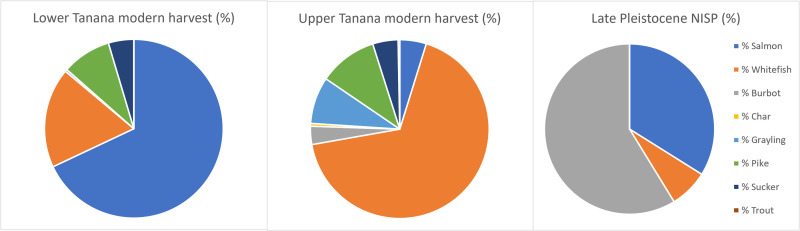
Comparison of combined late Pleistocene fish NISP with recent fish harvest (percent by taxon) from lower and upper Tanana Indigenous communities. Modern subsistence harvest data are from (*48*). Anadromous salmon are present in the lower Tanana but not the upper Tanana. The lower Tanana includes the 1984 harvest from Minto; the upper Tanana includes the combined 1987 harvest from Northway, Tetlin, and Tanacross. Trout (including stocked species) are harvested in the modern communities but at <0.5%; pike are present in the Pleistocene but at <0.5%.

While the few fish taxa observed (*n* = 4) are found at multiple sites, relative fish abundance is radically different among our samples. The vast majority of burbot and salmon specimens are found at Mead C3 and Upward Sun River C3, respectively, where they dominate each assemblage. This indicates the likely effects of seasonal exploitation of fish taxa, consistent with recent subsistence data on seasonal fishing (see the Supplementary Materials). In comparing these early fishing patterns with well-documented recent ethnographic fish use, we see that all observed late Pleistocene fish taxa are commonly harvested today. However, marked differences are apparent, with an overabundance of burbot and underabundance of whitefish in the late Pleistocene compared to the present (Fig. 3 and see the Supplementary Materials) (*48*).

These differences could be caused by multiple factors: (i) recovery bias, (ii) effects of climate and hydrological systems change on resource abundance, and/or (iii) differential human subsistence strategies and preferences. We reject recovery bias because each component was excavated using similar methods, the sediments from which the fish remains were recovered were broadly similar (aeolian loess deposits) (*49*, *50*), and the average maximum dimension for all identified taxa ranged from 4 to 9 mm, well above the screen mesh size used (3.175 mm). At Lime Hills Cave, in interior Alaska, smaller fish were identified in mixed natural/cultural contexts, including suckers, char, and grayling (*29*). Last, well-preserved small mammal and avian assemblages have been recovered in cultural contexts throughout the period of interest, 14,000 to 7000 cal yr B.P., suggesting that taphonomic effects may not have substantially affected observed fish presence and abundance.

While our knowledge of early natural fish abundance in Beringia is very limited, this study provides an initial relevant dataset. Salmon and whitefish are the most commonly used fish in the recent period, and the presence of both in the late Pleistocene suggests some level of continuity in available fish in the region. The absence of early grayling, char, and sucker may be due to variations in natural abundance or small size (~0.32 kg dressed weight compared to >0.9 to 2.450 kg for recovered taxa), but we note that ethnographic and modern subsistence data show that all three species are not commonly used (see the Supplementary Materials).

Estimating natural abundance of fish taxa in the deep past is difficult. Burbot and pike populations likely occupied freshwater refugia in western and central Beringia through the Last Glacial Maximum and dispersed to the east as deglaciation created suitable connected habitat (*51*, *52*). Salmon were likely available throughout interior eastern Beringia during the terminal Pleistocene, as ocean migration corridors were open. However, ocean environments and productivity were probably less optimal than today, and so lower abundance might have been expected during the full glacial, possibly increasing as conditions improved, perhaps as early as ~17,000 cal yr B.P. (*53*). A lake core δ^15^N record from central Alaska that begins ~15,000 cal yr B.P. as the site deglaciated suggests increasing salmon abundance between ~12,900 and 10,900 cal B.P. (*54*). At Lime Hills Cave in the Kuskokwim drainage, we identified five chum salmon through genetic analysis from stratum 3 in a mixed paleontological/cultural context, dating perhaps not only to the terminal Pleistocene but possibly also to the early Holocene (*20*, *29*).

No comparable estimates for Pleistocene freshwater fish taxa abundance are available; however, paleohydrologic changes in interior Alaska during this period have been developed from lake sediment records reconstructing lake levels and other proxies (*55*–*57*). These records suggest that many lakes were low or dry before ~14,000 cal yr B.P., with levels generally rising with some fluctuation into the mid-Holocene, due to increasing precipitation. Changes in vegetation from lake core pollen analysis track paleohydrologic proxies (*58*). Large interior lakes (e.g., Birch, Harding, and Quartz) with potential freshwater fish habitats were established between 16,400 and 13,500 cal yr B.P. (*58*, *59*), but Quartz Lake (near Cook site) was formed later at 11,200 cal yr B.P. (*60*), possibly affecting local whitefish and burbot presence. In any event, other archaeological sites in our sample are not located near substantial lakes (Fig. 1).

The temporal distribution of early fishing coincides with the Younger Dryas chronozone and the Betula Pollen Zone in the region (*61*), suggesting potential climatic drivers, either through changes in local hydrological systems or through human responses to changing climate and effects on local abundance of high ranked terrestrial prey. Some interior Alaska records of the Younger Dryas indicate drier conditions and an increase in *Artemisia* (*62*, *63*). These environmental changes suggest shifts in total fish habitat availability and likely relative changes in the proportion of stream-to-lake habitats. During the more arid Younger Dryas, it is possible that smaller, non-glacial streams were less suitable relative to lakes, perhaps explaining higher relative abundances of burbot to whitefish. For example, some lakes in the interior were closed basins without surface outlets during deglaciation, and outlets were established as lake levels rose into the Holocene (*55*). Increasing precipitation and associated vegetation changes during this period suggest substantial changes in terrestrial productivity that likely resulted in higher carrying capacity for some larger mammals into the Holocene, which may have influenced subsistence strategies. Previous faunal research in the region suggests a widening of diet breadth as a response to the onset of the Younger Dryas (*23*). Notably, more ameliorated periods before and after the Younger Dryas typically do not contain fish remains. The cause of the lack of early-middle Holocene fishing is unclear and may relate to changing natural fish abundance and/or a narrowing of diet breadth in favor of higher ranked megafauna (e.g., bison and wapiti) (*35*, *64*).

There are no transitions in regional cultural traditions that conform to the fishing/nonfishing patterns observed, suggesting that late Pleistocene fishing does not reflect a cultural adaptation of an incoming population. The earliest securely dated human populations in the Tanana, representing the Eastern Beringian tradition (~14,200 to 13,500 cal yr B.P., cultural periodization follows (*42*) apparently did not exploit freshwater or anadromous fish, and the Chindadn complex (~13,500–12,000 cal yr B.P.) spans the Allerød (with no fishing) and Younger Dryas (with fishing) chronozones. The Denali complex (~12,000–6000 cal yr B.P.) contains evidence of fishing during the Pleistocene/Holocene transition but little evidence of fishing after ~11,500 cal yr B.P.

There are marked differences between modern fish harvest patterns (*48*, *65*–*71*) and the Pleistocene fish record (Fig. 2). Ethnographic studies show that whitefish make up the bulk of freshwater fish subsistence harvest, while burbot comprise a small fraction. Ethnographic patterns of freshwater fishing indicate intense seasonal exploitation, a rapid start in April, peak harvests in the summer (mainly June–August) and a gradual decline in harvests during the fall (late August–November), with very little fishing in the winter and early spring (see the Supplementary Materials and fig. S1). Burbot are distinct from whitefish (and salmon) in that they are harvested in multiple seasons (but with lower quantities), including late winter and spring. Grayling and pike not only appear in much lower quantities throughout the summer but are also harvested in the spring and late fall, respectively. Salmon and whitefish have been critical resource staples for Indigenous communities in historic and modern periods, and summer communal fish camps were placed in key locations to intensively harvest, process (some dried), and store for the winter/early spring (*65*–*69*). These fish are almost exclusively caught in net/traps (often in conjunction with weirs) or more rarely with spears/leisters, but not with hook and line and almost never under the ice. Burbot, pike, and grayling are harvested in both nets/traps and hook and line, and the first two taxa are commonly caught under the ice in the fall (*65*, *66*, *69*–*71*). Fish wheels and rod and reel are in common use today but are recent introductions to the region from the early 1900s (*65*).

In contrast, late Pleistocene freshwater and anadromous fish record suggests different fish harvesting strategies. None of these early sites can be characterized as dedicated fish camps, and all are dominated by mammalian resources, suggesting their use as hunting, transitory, or residential base camps. These ancient peoples used the same fish species as regional Native peoples today but with different foci, likely conditioned by local seasonal abundance fluctuations, e.g., summer salmon at Upward Sun River C2, C3, and XBD-318 and spring burbot at Mead C3. We can characterize late Pleistocene fishing as more situational than the intensive bulk harvesting typical of salmon (and whitefish) exploitation in the ethnographic record, given the similar low levels of abundance and body part representation seen at Upward Sun River C3 and Mead C3. Freshwater and anadromous fish formed important elements of a broad-spectrum diet that also incorporated small terrestrial mammals and birds, waterfowl, and large game (e.g., bison, and wapiti) (*23*, *72*). This interpretation is supported by the comprehensive isotopic paleodiet analysis of two 11,500 cal yr B.P. infants at Upward Sun River C3, indicating mothers’ summer diets of ~65% terrestrial, 30% salmon, and 5% freshwater resources (*18*). We see no evidence of intensive processing and/or storage of fish (e.g., no cache pits during this period), suggesting local consumption during the course of short-term site occupations. Similarities in body part representation between burbot (not historically captured in bulk and stored) and salmon additionally suggest similar low levels of exploitation for both species. A more direct measure of Younger Dryas salmon exploitation is provided by serial sampling of an Upward Sun River individual’s dentition indicating a measurable increase in salmon diet of the mother between spring and summer, providing evidence against overwinter caching of salmon (*18*).

While organic tool preservation is relatively common in these middle Tanana River basin sites (*23*, *42*, *73*), no clear evidence of fishing technology has been identified (e.g., fishhooks, leisters, and net weights). The archaeological and ethnographic patterns for the region suggest that small-scale net technology (e.g., dip and set/gill nets) was likely used in the late Pleistocene, given that (i) hooks, leisters, and barbed points would have undergone more curation, are made of durable materials, and would be preserved if present; (ii) some nets (e.g., dip nets) do not require net sinkers; (iii) nets have been inferred for early Eurasian Paleolithic peoples, potential ancestors of Paleoindians (*74*), and net use has been inferred in early Beringian sites (*16*, *75*); and (iv) nets/traps (often in conjunction with weirs) were by far the most common fish capture technology among historic Indigenous peoples in the region (*65*, *66*, *68*–*71*).

This record can also be used to understand Paleoindian lifeways in Beringia. The earliest peoples (>13,000 cal yr B.P.) did not regularly exploit freshwater or anadromous fish but focused on terrestrial megafauna, supplemented with smaller game and waterfowl (*23*, *72*, *76*), consistent with models of Paleoindian ecology with high residential mobility associated with megafaunal hunting (*77*). However, during the Younger Dryas, possibly as a response to declines in regional megafauna (and perhaps waterfowl) abundance, ancient Alaskans expanded their diet breadth and began to harvest reliable and predictable anadromous and freshwater fish. This suggests that the earlier high-tech forager strategy was replaced by more collector-like strategies [sensu Binford (*78*)], implying somewhat reduced mobility, local knowledge of seasonal fish abundance, and location reflecting a mapping-on strategy, with fish likely being a secondary factor in site location, particularly of residential base camps (such as Upward Sun River C3 and Mead C3) where longer-term and broader spectrum central place foraging strategies would be expected. In expected periods of resource stress, e.g., late winter/early spring (*68*), seasonal exploitation of various fish taxa may have been critical as a buffer food. During the early Holocene, fishing lessened, perhaps due to changing environments favoring increasing bison and wapiti abundance (*64*). Beyond freshwater and anadromous fishing described here, we note that the earliest secure evidence of maritime fishing in North America dates after the onset of the Younger Dryas, ~11,800 to 11,500 cal yr B.P. (*79*), suggesting continent-wide broader changes in economies, perhaps also in response to climate change, resource abundance changes, and local adaptations.

Rather than a simple long-term economic intensification and shift from forager to collector economic strategies, the variation in early human fishing in Beringia suggests more complicated patterns of resource use in response to long-term environmental changes. Our data collectively suggest that changes in climate and ultimately key mammal resources during the Younger Dryas led to human responses of widening diet breadth to incorporate multiple species of freshwater and anadromous fish, setting a pattern that would be expanded upon later in the Holocene as fish, particularly salmon, became key resources to Alaska Native lifeways.

## MATERIALS AND METHODS

### Archaeological sampling

All archaeological samples were recovered from sites with components encased in loess (wind-blown silt) deposits that range in 0.9 to more than 3 m in thickness. Our archaeological methods at each site follow standard practices including screening sediments with a 1/8^″^ screen mesh and 100% collection of bulk sediment samples of cultural features (e.g., hearths) to float lighter materials and water screen through finer mesh sizes. We provide descriptions for each site included in this study, along with brief summaries of local geomorphological, ecological, and hydrological settings in the Supplementary Materials.

### Zooarchaeological analyses

Fish specimens were identified to the lowest taxonomic level possible with the aid of a fish skeletal comparative collection following diagnostic criteria described in Choy *et al.* (*19*). Specimens were included only if they could be identified without the aid of magnification. We use the term “salmon” to refer to all remains identified to the genus *Oncorhynchus*, because no trout forms of this genus (e.g., *Oncorhynchus mykiss*) are native to the Tanana basin. Salmon native to the region include *O. keta* (chum), *Oncorhynchus kisutch* (coho, silver), and *Oncorhynchus tshawytscha* (Chinook, king). “Salmonid” refers to any member of family Salmonidae, which can include freshwater and anadromous taxa; in the Tanana Valley, salmonids include *Oncorhynchus* spp. (salmon), the subfamily Coregoninae (whitefish), *T. arcticus* (grayling), and *Salvelinus* spp. (char). The term “unidentified fish” refers to all specimens that could be assigned to Actinopterygii, but that could not be assigned to a lower taxonomic level.

Skeletal element, side (if applicable), element portion, and surface modifications were recorded where possible. Standard taxonomic abundance measures were calculated, including NSP and NISP (*80*). Here, NSP includes all specimens (identified and unidentified), while NISP includes the specimens that were identified to at least order. Taphonomic information was recorded for each specimen, including degree of burning and bone completeness. In addition, we used two indices to assess identifiability and fragmentation, including NSP/NISP [“the number of bones examined to yield one identified specimen” (*30:1173*)], and the identification rate (NISP/NSP × 100) (*81*).

### Stable isotope analysis

All but one of the fish bone collagen extractions were conducted at the Laboratory of Environmental Archaeology, University of Alaska Fairbanks using a modified Longin protocol described previously (*17*). Carbon and nitrogen stable isotope measurements on these samples were made on a Thermo Finnigan Delta Plus XP continuous flow isotope ratio mass spectrometer, coupled to a Costech elemental analyzer (ECS 4010), at the Washington State University Stable Isotope Core Laboratory. The stable isotope compositions were calibrated relative to Vienna Pee Dee Belemnite (VPDB) (carbon) and AIR (nitrogen) using at least two internal standards, which had been previously calibrated against internationally certified standards. Precision, calculated as the pooled SD of all repeated measures of calibration and check standards (*82*) was 0.14‰ for both ^13^C and ^15^N. All bone collagen samples analyzed at Washington State University met accepted quality standards: %N > 5%, %C > 13%, an atomic C/N ratio of 2.9 to 3.6, and a collagen yield of >1%. One bone collagen sample (ID 3837) was extracted and analyzed by the Center for Applied Isotope Studies at the University of Georgia. This extracted collagen sample was too small to allow for nitrogen stable isotope analysis.

### DNA taxonomic identification

DNA taxonomic identification was attempted for a limited number of previously unpublished fish specimens (*n* = 16). Analyses were conducted at Washington State University and the University of Oklahoma.

### Washington State University

All pre–polymerase chain reaction (PCR) activities were conducted in the ancient DNA (aDNA) laboratory at Washington State University. This laboratory, located in a separate building from wherein PCR and post-PCR activities are conducted, is a dedicated workspace for processing aged, degraded, and/or low copy number DNA samples. Precautions aimed to minimize and monitor the introduction of contamination are practiced in the laboratory.

#### DNA extraction method 1

Approximately 20 to 51 mg of bone material were subsampled from specimens UAF-01 to UAF-10 (case numbers 3384, 3428, 3438, 3457, 3519, 3724, 3732, 3745, 3836, and 3997). The subsamples were submerged in 6% (w/v) sodium hypochlorite for 4 min (*83*). The sodium hypochlorite was poured off, and the samples were quickly submerged in DNA-free water twice. The bone subsamples were transferred to 1.5-ml tubes, to which aliquots of 500 μl of 0.5 M EDTA were added, and the tubes gently rocked at room temperature for >48 hours. An extraction negative control, to which no bone material was added, accompanied this batch of extractions. These controls are tested alongside the samples with PCR (described below) to help us determine whether the contamination was introduced during the extraction.

DNA was extracted following the method described by Kemp *et al.* (*84*). Ninety microliters of proteinase K (BIOBASIC, catalog no. 32181) at a concentration of 1 mg/30 μl (or >20 U/30 μl) was added to each sample, and the tubes were incubated at 64° to 65°C for 3 hours. Following proteinase K digestion, the tubes were centrifuged at 15,000 rpm for 1 min to pellet any undigested bone, dirt, and/or “sludge.” The volumes of liquid were moved to new 1.5-ml tubes, to which 750 μl of 2.5% “resin” (i.e., 2.5% celite in 6 M guanidine HCl) and 250 μl of 6 M guanidine HCl were added. The tubes were vortexed multiple times over approximately a 2 min period.

Promega Wizard minicolumns were attached to 3-ml Luer-Lok syringe barrels (minus the plunger) and placed on a vacuum manifold. Three millilters of DNA-free water was first pulled across the columns with the intent to wash away potential contaminating DNA. The DNA/resin mixture was subsequently pulled across the columns. The silica, now pelleted on the minicolumns, was then rinsed by pulling 3 ml of 80% isopropanol across the columns.

The minicolumns were then placed in new 1.5-ml tubes and centrifuged at 10,000 rpm for 2 min to remove excess isopropanol. The minicolumns were transferred to new 1.5-ml tubes. Fifty microliters of DNA-free water heated to 64° to 65°C were added to the minicolumns and left for 3 min before centrifugation of the tubes for 30 s at 10,000 rpm. This step was repeated, amounting to 100 μl of extracted DNA. Ten microliters each of the full concentration eluates and extraction negative control was diluted 1:10 and 1:50 with water and used in PCR, as described below.

#### DNA extraction method 2

Approximately 33 to 177 mg of bone material were subsampled from specimens UAF-01 (case number 3384) and UAF-02 (case number 3428) and UAF-04 to UAF-10 (case numbers 3457, 3519, 3724, 3732, 3745, 3836, and 3997) (the material from sample UAF-03 was exhausted following the extraction method 1).

The bone subsamples were transferred to 15-ml tubes, to which aliquots of 2 ml of 0.5 M EDTA were added, and the tubes gently rocked at room temperature for >48 hours. An extraction negative control, to which no bone material was added, accompanied this batch of extractions.

DNA was extracted following a modified Kemp *et al.* (*85*) method described by Moss *et al.* (*86*). Ninety microliters of proteinase K (BIOBASIC, catalog no. 32181) at a concentration of 1 mg/30 μl (or >20 U/30 μl) was added to each sample, and the tubes incubated at 64° to 65°C for 3 hours. DNA was first extracted by adding an equal volume of phenol:chloroform:isoamyl alcohol (25:24:1) to the EDTA, and the tubes were rocked gently for 5 min and then centrifuged at 3100 rpm for 5 min. The aqueous phases were moved to new 15-ml tubes, and the DNA was subsequently extracted using phenol:chloroform:isoamyl alcohol (25:24:1), as just described. A third extraction was performed using an equal volume of chloroform:isoamyl alcohol (24:1), and the tubes were gently rocked for 5 min and then centrifuged at 3100 rpm for 3 min. The aqueous phases were moved to new 15-ml tubes.

DNA was precipitated from solution by adding one-half volume of room temperature 5 M ammonium acetate and, to this combined volume, one volume of room temperature absolute isopropanol (*87*). This mixture was stored overnight at room temperature. Centrifuging the tubes for 30 min at 3100 rpm pelleted the DNA. The isopropanol was carefully poured off, and the inverted tubes were air-dried for 15 min. The DNA pellets were washed with 1 ml of 80% ethanol with vortexing. Centrifuging the tubes for 30 min at 3100 rpm again pelleted the DNA. The ethanol was decanted, and the inverted tubes were air-dried for 15 min.

The DNA pellets were resuspended in 300 μl of DNA-free water, and the volumes were transferred to new 1.5-ml tubes. To these volumes, 750 μl of 2.5% “resin” (i.e., 2.5% celite in 6 M guanidine HCl) and 250 μl of 6 M guanidine HCl were added. The silica-based extraction then followed that described in the “DNA extraction method 1” section.

#### Inhibition test and repeat silica extraction

The full concentration DNA eluates and the extraction negative controls were tested for the presence of PCR inhibitors following the rationale of Kemp *et al.* (*84*) using a “goose collective” as the aDNA positive control (see their figure 1). The DNA recovered from several archaeological Aleutian cackling goose (*Branta hutchinsii leucopareia*) bones (*88*) was pooled together to make the goose collective. The choice to pool these individual extractions was made with the intention to reduce variance between goose DNA eluates in both endogenous mitochondrial DNA (mtDNA) copy number and possible inhibitors co-extracted with the goose DNA. Before they are used in experiments, each goose collective was demonstrated to PCR amplify consistently (in six or more amplifications), hence serving as a positive control.

PCRs (15 μl) were conducted to amplify a 159–base pair (bp) portion of goose mitochondrial cytochrome B gene using the primers “BSP-I” and “GooseR” described by Wilson *et al.* (*88*). The components of these PCRs were as follows: 1X Omni Klentaq reaction buffer (including a final concentration of 3.5 mM MgCl_2_), 0.32 mM deoxynucleotide triphosphates (dNTPs), 0.24 μM each primer, 0.3 U of Omni Klentaq LA polymerase, and 1.5 μl of goose collective DNA. These reactions were spiked with 1.5 μl of potentially inhibited, full concentration DNA eluates recovered from the samples under investigation here. These PCRs were run in parallel with reactions that contained only goose collective DNA (i.e., were not spiked). These reactions served as positive controls and allowed us to preclude PCR failure from contributing to our results. PCR negatives also accompanied each round of amplification, allowing us to monitor for possible contamination. Following denaturing at 94°C for 3 min, 60 cycles of PCR was conducted at 94°C for 15 s, 60°C for 15 s, and 68°C (note that this is the optimal extension temperature for Omni Klentaq LA polymerase) for 15 s. Last, a 3-min extension period at 68°C was conducted before bringing the reactions to 10°C.

If the goose collective failed to amplify when spiked with any given aDNA eluate, then we considered the eluate to be inhibited. In the case that spiking the aDNA permitted amplification of the goose collective DNA, we consider that DNA eluate to be inhibitor “free.”

Full concentration eluates deemed to be inhibited in this manner were subjected to repeat silica extraction (*84*). To the remaining volume of the eluate, 750 μl of 2.5% resin and 250 μl of 6 M guanidine HCl were added. The samples were vortexed numerous times over a 2-min period. The silica-based extraction then followed that described under the “DNA extraction method 1” section, except that the volume used to elute the DNA from column matched the volume being repeat silica extracted. For example, if the starting volume was 97 μl, 48.5 μl of DNA-free water heated to 65°C would be added to the minicolumns and left for 3 min before centrifugation. This step would be repeated twice for a total volume of 97 μl.

These repeat silica eluates were tested again for inhibition as described above. Those still deemed to be inhibited were once again repeat silica extracted and tested again for inhibition. This was carried out until all full concentration eluates were deemed to be uninhibited.

#### Fish species identification and PCR

For fish species identification, we used primers previously described by Jordan *et al.* (*89*) that amplify 189 bp from the mitochondrial 12*S* gene [relative to a rainbow trout (*O. mykiss*), reference sequence (DQ288271.1)]. While the sequences produced with these primers were designed specifically to allow one to discriminate between species of Pacific salmonids, they are also capable of amplifying mtDNA from a wide range of fish, including even cartilaginous species (*90*). Note that Jordan *et al.* (*89*) originally described their reverse primer in the wrong orientation. The corrected primers are OST12S-F (5′-GCTTAAAACCCAAAGGACTTG-3′) and OST12S-R (5′-CTACACCTCGACCTGACGTT-3′).

Full concentration eluates (deemed to be uninhibited) and the 1:10 and 1:50 dilutions of the original full concentration DNA eluates from the “DNA extraction method 1” section and the full concentration eluates (deemed to be uninhibited) from the “DNA extraction method 2” section were subjected to PCR amplification as follows. First, “standard” PCRs contained 1× Omni Klentaq reaction buffer, 0.32 mM dNTPs, 0.24 μM each primer, 0.3 U of Omni Klentaq LA polymerase, and 1.5 μl of template DNA. Second, rescue PCR at a 25% increase was carried out as described by Johnson and Kemp (*91*). Rescue PCRs contained 1.25× Omni Klentaq reaction buffer (including a final concentration of 4.375 mM MgCl_2_), 0.4 mM dNTPs, 0.3 μM each primer, 0.375 U of Omni Klentaq LA polymerase, and 1.5 μl of template DNA. For both forms of PCR, denaturing at 94°C for 3 min was followed by 60 cycles of PCR conducted at 94°C for 15 s, 60°C for 15 s, and 68°C for 15 s. Last, a 3-min extension period at 68°C was conducted before bringing the reactions to 10°C. PCR negatives accompanied batches of amplification to allow us to monitor for the presence of contaminating DNA. Positive controls of sockeye salmon (*Oncorhynchus nerka*), added in the PCR laboratory just before running the reactions, accompanied all PCRs to monitor for possible failure.

### University of Oklahoma

All pre-PCR activities were conducted in the aDNA facility at the Laboratories of Molecular Anthropology and Microbiome Research (lmamr.org) at the University of Oklahoma. This facility is a dedicated workspace for processing aged, degraded, and/or low copy number DNA samples. Precautions aimed to minimize and monitor the introduction of contamination are practiced in the laboratory.

#### DNA extraction

DNA was extracted from approximately 28.1 to 45.1-mg subsamples of 16-233, 17-265, 17-266, 17-267, 17-268, and 17-269 (case numbers 4137, 30, 32, 33, 34, and 31, respectively) following the “DNA extraction method 1” section, as described above under Washington State University methods. Ten microliters of the full concentration eluates and extraction negative controls was diluted 1:10 with water and used in PCR, as described below.

#### Inhibition test and repeat silica extraction

Full concentration DNA eluates were evaluated for inhibition as described under the Washington State University methods with the following modifications. The aDNA control in this case was DNA pooled from that extracted from various archaeological turkey (*Meleagris gallopavo*) bones (*92*). Fifteen–microliter inhibition test PCRs amplified a 186-bp portion of turkey displacement loop using the primers “T15709F” and “T15894R.” Aside from using different primers, the other components of these PCRs and cycling condition were identical to those described under the Washington State University methods. Positive controls of turkey, added in the PCR laboratory just before running the reactions, accompanied all PCRs as an additional means to monitor for possible PCR failure. PCR negatives also accompanied each round of amplification, allowing us to monitor for possible contamination.

#### Fish species identification and PCR

Fish species identification was conducted as described under the Washington State University methods. All full concentration eluates (deemed to be uninhibited) and 1:10 dilutions of the original eluates were amplified using standard PCR and rescue PCR. Furthermore, we also used a PCR enhancer cocktail called PEC-P (DNA Polymerase Technology) in additional PCRs. This enhancer has been found to be useful in the amplification of aDNA (*90*, *93*). Fifteen microliters PEC-P PCR reactions contained 1× Omni Klentaq reaction buffer, 0.32 mM dNTPs, 0.24 μM each primer, 0.3 U of Omni Klentaq LA polymerase, 20% (v/v) PEC-P, and 1.5 μl of template DNA. PCR cycling conditions for PEC-P PCRs were same as those for standard and rescue PCR as described above under the Washington State University methods. PCR negatives accompanied batches of PEC-P amplification to allow us to monitor for the presence of contaminating DNA. Positive controls of sockeye salmon, added in the PCR laboratory just before running the reactions, accompanied all PCRs to monitor for possible failure.

### Additional analyses of samples extracted at Washington State University

The UAF series of DNA eluates and their dilutions were moved to the University of Oklahoma. Of those samples processed in the “DNA extraction method 1” section, the full concentration DNA eluates (deemed to be uninhibited) and the 1:10 and 1:50 dilutions of the initial eluates were subjected to PEC-P PCR. Full concentration DNA eluates (deemed to be uninhibited) from the “DNA extraction method 2” section were also subjected to PEC-P PCR. The eluates in the “DNA extraction method 2” section and their 1:10 dilutions (made at the University of Oklahoma) were subjected to standard PCR.

## Supplementary Materials

### This PDF file includes:

Supplementary Text Fig. S1 Tables S1 to S8 Legend for dataset S1 References
