# Supplementary material for: Freshwater and anadromous fishing in Ice Age Beringia
Source: Sci Adv. 2023 Jun 2;9(22):eadg6802. doi: 10.1126/sciadv.adg6802 (PMC10413661; doi:10.1126/sciadv.adg6802)
Supplement: Supplementary file 1 — Supplementary Text Fig. S1 Tables S1 to S8 Legend for dataset S1 References [file sciadv.adg6802_sm.pdf]

Science Advances
AAAS

Supplementary Materials for

Freshwater and anadromous fishing in Ice Age Beringia

Ben A. Potter *et al.*

Corresponding author: Carrin M. Halffman, cmhalffman@alaska.edu; Ben A. Potter, rhovanion25@gmail.com

Sci. Adv. **9**, eadg6802 (2023)
DOI: 10.1126/sciadv.adg6802

The PDF file includes:

Supplementary Text
Fig. S1
Tables S1 to S8
Legend for dataset S1
References

Other Supplementary Material for this manuscript includes the following:

Dataset S1

Supplementary Text

1. Site Descriptions

All archaeological samples were recovered from sites with components encased in loess (wind-blown silt) deposits that range in 0.9 m to over 3 m in thickness. Our archaeological methods at each site follow standard practices including screening sediments with a 1/8" screen mesh and 100% collection of bulk sediment samples of cultural features (e.g., hearths) to float lighter materials and water screen through finer mesh sizes. Below is a description for each site included in this study, along with brief summaries of local geomorphological, ecological and hydrological settings.

1.1. Cook Site. The Cook Site (49XBD-072) is situated on a relatively flat surface of the toe of a bedrock ridge that overlooks the southeastern edge of the Shaw Creek Flats to the north, as well as the Tanana River to the west. The current course of the Tanana River is located less than 800 m from the site and the toe of the ridge rises between 8 to 15 m above the surrounding floodplain. Little Lost Lake and Quartz Lake are within 3 km of the site. The site has between 100 and 300 cm of aeolian sand and silt (loess) deposits that cover the bedrock (*49*). A series of occupations are associated with at least 11 buried soils and soil complexes that date between 1960±30 BP (AA-105232) and 10,400±60 BP (AA-106445) (*96*). A series of 6 thicker buried soils with more developed horizonation are present between 40 and 100 cmBS and represent incipient forest soils dating back to 7,850+/-50 B.P. (AA96873) (*49*). Soils and soil complexes below 100 cmBS generally consist of very weakly developed, thin (less 1 cm thick) organic and carbonate-rich silt loams likely associated with more herbaceous tundra vegetations (*49, 96*). Pike remains at the site are associated with the lowest component and the 10,400±60 BP date on a fragment of large mammal bone (*15*).

1.2. Hollembaek's Hill Site. The Hollembaek's Hill Site (49XBD-376) is located southside of a bedrock outcrop that overlooks the plain of the Tanana River to the north and east and sand dunes to the south and west. The Tanana River is constricted against the Yukon-Tanana Uplands in this area but has likely changed its course numerous times in the low-lying portions of the plain since the Late Pleistocene and the earliest human occupation of the site. The main course of the Tanana is about 760 m and Volkmar Lake is 7.5 km to the north of the site. Volkmar Lake contains pike *Esox lucius*), humpback whitefish (*Coregonus pidschian*), least cisco (*Coregonus sardinella*), and slimy sculpin (*Cottus cognatus*) (*97*). The stratigraphy in the main area of the site is comprised of 170-210 cm of loess deposits overlying the bedrock. Archaeological occupations are associated with at least 9 paleosols that date as early as 11,770±30 BP (UGAMS-43628) (additional dates in *45*). All fish remains at the site were recovered within or near an anthropogenic feature dated to ca. 7020-7690 ^{14}C BP (~8800-7890 cal BP), itself part of an Early Holocene archaeological component otherwise dated to 7530 ^{14}C BP (*45*). Two fish remains were dated directly: one salmon specimen to 7920±25 BP (UGAMS-43627), uncorrected for reservoir effect, and one whitefish specimen to 9320±30 BP (UGAMS-32232) (10,650-10410 cal yr BP). The discrepancy between these two dates and the other dates in the feature are likely due to a radiocarbon reservoir effect, the intensity of which is poorly known in the Tanana watershed. However, extensive anthropogenic and biogenic disturbance in this area of the site makes it possible (if unlikely given

the absence of fish remains elsewhere at the site) that some of the fish remains are from older contexts.

1.3. Mead. The Mead Site (XBD-071) is situated on a loess mantled bedrock knob directly overlooking the Shaw Creek Flats, with Shaw Creek currently 460 m from the site. It was tested in 1990, 1992 by Holmes and full-scale excavations were conducted in 2009, 2011-2014, 2016 by Potter (*23, 46*). Site geoarchaeology has been previously described by Dilley (*50*), Gilbert (*98*) Potter et al. (*46*). A total of 287 m^2 has been excavated, yielding 8 components dating from the Bolling (C1) to the late Holocene, around 3000 cal yr BP (C6). Fauna are well preserved, and 39,137 faunal fragments have been recovered, along with 35,160 debitage, 307 tools, and 35 hearth features (*23, 98, 99*). The site is well stratified, with 5 components associated with Ab horizons between 13,440-11,850 cal yr BP (*23*). These early components exhibit very minimal post-depositional disturbances (*46, 98*). The fish remains reported here derive from two components: C2b, C3a. Component C2b is a Chindadn tradition occupation at ~110-120 cmBS, containing 8 hearths, 2,810 lithics including 52 tools, and 3,254 bone fragments. Four of the hearths are dated yielding overlapping ages between 13,110-12,790 cal yr BP. Faunal remains from C2b include *Bison priscus* (steppe bison), *Cervus canadensis* (wapiti), and waterfowl (*23*). Component C3a is a Denali tradition occupation at ~90-100 cmBS, containing 14 hearths, 8,483 lithics including 92 tools and over 28,000 bone fragments. Seven dated hearths have overlapping ages between 12,200-11,820 cal yr BP. Faunal remains from C3 include *Bison priscus* (steppe bison), *Alces alces* (moose), *Lepus* sp. (hare), *Urocitellus parryi* (ground squirrel), Canidae (canids) and waterfowl (*23*). It is important to note that four other components (C1, C2a, C3b, and C4) date to about the same periods (between 13,480 and 11,820 cal yr BP) have similar depositional conditions, and contain fauna, but do not contain fish remains.

1.4. Swan Point. Swan Point (49XBD-156) was discovered in 1990 and has been under investigation intermittently since that time (*40, 100*). The site is located approximately 3.5 km north of Shaw Creek and 6.5 km north of the Tanana River on a small hill in the Shaw Creek valley, a boggy area with small streams, ponds, and patches of the boreal forest. Major excavations took place in 2003, 2005, and 2013 (*23, 43*). Beginning in 2014 the Swan Point investigations have been carried out under the Shaw Creek Archaeological Project, a long-term multidisciplinary project, at the Alaska Museum of the North, University of Alaska. Approximately 100 m^2 have been excavated to date. Eight components have been identified that document occupation at the site from the terminal Pleistocene to the historic period (*42, 43, 101*). Burbot remains, dated to 11,210 ± 35 BP (UGAMS-26402: 13,170-13,090 cal yr BP), were recovered between two late Pleistocene archaeological traditions: 1) the East Beringian tradition, Diuktai phase, CZ4b, at c. 14,200 cal yr BP, supported by a dozen radiocarbon dates (*102*); and 2) the Chindadn tradition, Phase 1, CZ3b, that has a date range on four hearths between c. 12,710 and 12,620 cal yr BP. Fauna recovered at this early period includes mammoth, horse, bison, wapiti, caribou, canid sp., along with swans, geese, and small mammals (*15, 45, 76*). Lithic tool analyses (*102, 103*) link Swan Point to Late Pleistocene cultures in eastern Siberia.

1.5. Upward Sun River. The Upward Sun River Site, or Xaasaa Na’ (49XBD-298), discovered in 2006, is situated in a loess-mantled sand dune complex near the edge of an alluvial terrace about 4 km south of the current course of the Tanana River (*104*). Small sloughs are present directly below the alluvial terrace a few hundred meters north of the site. The site was tested in

2007 and excavated in 2010-2011, 2013-2014, with a total of 240 m^2 excavated. Upward Sun River geoarchaeology and site formation have been previously reported (*32, 104*). A series of 27 radiocarbon dates secure six components, dating from the Bølling-Allerød period to the late Holocene. The fish remains reported here derive from Component 3, a Denali tradition occupation ~90-100 cmBS, associated with 10 hearths and a burial pit dating to ~11,500 cal yr BP. A female infant and a female neonate were interred in a burial pit, and a 3-year old was cremated within an overlying hearth, all occurring within a short time during the summer (late July – early August) (*32, 104*). Several thousand lithics, including 73 tools (projectile points, bifacial knives, unifacially retouched flakes) and 7,691 debitage are directly associated with the features and 930 Number of Identified Specimens (NISP) of fauna, primarily found within a cremation hearth and burial pit fill (*32*). The most abundant taxa are *Oncorhynchus* sp. (salmon) (337 NISP), *Urocitellus parryi* (ground squirrel) (242 NISP) with small amounts of *Lepus americanus* (snowshoe hare), *Marmota* sp. (marmot), and Tetraoninae (ptarmigan/grouse) (*32*). Genetic and isotopic analyses of Upward Sun River fish indicated the earliest human use of salmon in the Americas (*17*). Chemical analyses of 9 Component 3 hearth feature sediments indicate relative intensity of resource use: 53% terrestrial, 22% anadromous salmon, and 24% freshwater resources (e.g., freshwater fish, waterfowl) (*19*). Recent paleodiet analyses indicated mothers' diets of 62-65% terrestrial, 30-32% anadromous salmon, and 5-6% freshwater resources, broadly consistent with the zooarchaeological, genetic, isotopic, and geochemical analyses (*18*). A direct 14C assay on chum salmon collagen yielded an age of 10,462+/-34 BP (UGAMS-30061) (12,620-12,110 cal yr BP), indicating a marine reservoir offset of ~490 years from the terrestrial charcoal record mean pooled average for Component 3 of 9970+/-30 BP (11,600-11,270 cal yr BP).

1.6. XBD-318. XBD-318 (49XBD-318), discovered in 2006, is situated on the narrow point of the northern edge of an alluvial terrace that rises 12-15 m above the valley floor and the surrounding muskeg flats (*41*). The current Tanana River lies 4.8 km north of the site, and the nearest water body are small sloughs and ponds within 500 north and below the XBD-318 terrace. Seventeen lithic artifacts and 16 fragmented animal bones (bird, fish, and small and medium mammals) were recovered between 50-108 cmBS from two 50 cm^2 test pits. The terrace's edge is 200-250 m northeast of a small lake, and roughly 4.5 km south of the Tanana River and west of Delta Creek, and 15.5 km east of the Little Delta River. The stratigraphy is comprised around 120 cm of alluvial and aeolian sand and loess overlying glaciofluvial gravels. Surficial soil development is between 30-35 thick. A single salmon vertebra was recovered from loess around 100-110 cmBS (*41*). A direct assay on collagen from this specimen yielded a date of 10,830±40 BP (UGAMS-26403) (12,826-12,729 cal yr BP); however this date does not include a correction for a marine reservoir effect (MRE). There are no systematic studies of MREs in North Pacific salmonids. Only a few studies quote MREs in salmonids; however, there were only two or less specimens within each of these studies. Southon and Fedje (*105*) presented salmon bones that are 970±60 years offset [R(t)] from terrestrial pairs in British Columbia; de Flamingh et al. (*106*) show an offset of 950±45 years for a salmon bone from wood paired samples on the Kenai Peninsula [see Schmuck et al. (*107*) for R(t) calculations for Southon and Fedje (*105*) and Flamingh et al. (*106*)]. Ledger et al. (*108*) quote a 700 year difference between salmon and caribou bones from the Bering Sea region. Assuming a ~960 year reservoir correction, the XBD-318 salmon dates to ~11,393-11,204 cal yr BP. The offset of a single directly dated salmon vertebra from Upward Sun River (10462+/-34 14C yr BP) yields an offset

of 492 years, which would yield a date of 12,465-11,946 cal yr BP. For the purposes of this paper, we interpret the reservoir corrected age to be ~12,000-11,300 cal yr BP.

2. Detailed DNA Results

2.1. Washington State University. Of those eluates produced with DNA Extraction Method 1, UAF-01, UAF-06, UAF-07, UAF-09, and UAF-10 (case numbers 3384, 3724, 3732, 3836, and 3997, respectively) required one round of repeat silica extraction each to sufficiently remove inhibition. All of the eluates produced with DNA Extraction Method 2 were deemed to be uninhibited and did not require repeat silica extraction. None of the full concentration eluates or their dilutions amplified with 12S fish primers. It is also notable that none of the extraction negative or PCR negative controls amplified.

2.2. University of Oklahoma. All of the eluates produced with DNA Extraction Method 1 were deemed to be uninhibited and did not require repeat silica extraction. With the positive results summarized below, it is notable that none of the extraction negative or PCR negative controls tested positive for fish mtDNA.

The sequence from sample 16-233 (case # 4137) matches 100% to a burbot (*Lota lota*) [aligned to nucleotide positions (nps) 592-740 of a full mitogenome reference (Genbank accession KM363244.1; (*109*)]. This sequence was observed six times, twice each using standard PCR with the full concentration eluate and the 1:10 dilution and also at full concentration and at 1:10 using PEC-P. This result matches the morphological identification of the specimen as a burbot.

Sample 17-265 (case # 30) was identified as a chum salmon (*Oncorhynchus keta*), following Jordan et al. (*91*). Aligned to nps 592-740 of a full rainbow trout (*Oncorhynchus mykiss*), mitogenome reference (Genbank accession DQ288271.1; (*110*)], chum salmon exhibit C660T and C713T mutations. This sequence was observed five times: 1) at full concentration using standard, rescue, and PEC-P PCR, and 2) at 1:10 using standard and PEC-P PCR.

Sample 17-266 (case # 32) was identified as a chum salmon from one sequence produced from the full concentration DNA eluate amplified with PEC-P PCR. A second sequence amplified with PEC-P from the 1:10 dilution exhibits the C660T and C713T mutations in the forward direction, however the reverse sequence failed. This second sequence, thus, spans nps 638-740. Regardless, this second sequence could not be mistaken for any other Pacific salmonid than a chum salmon [see Table 2 of Jordan et al. (*91*)].

Sample 17-267 (case # 33) was identified as a chum salmon from two sequences, one produced with standard PCR amplification from the 1:10 dilution and the second produced from amplification of the 1:10 dilution with PEC-P.

Sample 17-268 (case # 34) was identified as a chum salmon from three sequences, one produced with standard PCR amplification from the 1:10 dilution and one each from the full concentration DNA eluates subject to rescue PCR and PEC-P.

Sample 17-269 (case # 31) was identified as a chum salmon from four sequences: 1) one each from amplification of the full concentration eluate using standard, rescue, and PEC-P PCR, and 2) from amplification of the 1:10 dilution with PEC-P.

It is notable that samples 17-265, 17-266, 17-267, 17-268, and 17-269 were all assessed morphologically to be Pacific salmonid (*Oncorhynchus* sp). Previous studies have demonstrated that speciating salmon based morphology alone is difficult (*88*, *111*). The combination of approaches used here lends confidence that all of these specimens are of chum salmon.

Amplification with PEC-P from the full concentration eluate of UAF-04 (case # 3457) (produced from DNA Extraction Method 2 at Washington State University) yielded a sequence that most closely resembles burbot, according to a blast search of NCBI. The top seven hits are burbots, all with 98.66% identity. Aligned against the burbot full mitogenome reference (Genbank accession KM363244.1; Zhang (*109*)], this sample exhibits C607T and C608T mutations. This leads to a two possibilities. First, it is possible that both of these "mutations" are the product of post-mortem DNA damage, a common form of which is C to T "transitions", which is precisely what is observed here. Second, this sample could exhibit a burbot lineage that has previously not been previously observed. In either case, our inability to replicate this sequence does not allow us to reject either possibility. Consistent with morphological assessment of the specimen, this sample is likely a burbot.

The 1:10 dilution of UAF-06 (case # 3724) (produced from DNA Extraction Method 2 at Washington State University) amplified with standard PCR conditions. Following Jordan et al. (*91*), the sequence matches to Chinook salmon [exhibiting G629T, C660T, C711T, and G730A mutations relative the full rainbow trout mitogenome reference DQ288271.1 (*110*)]. However, morphological assessment of this specimen indicates that it is not a Pacific salmonid, but rather is a burbot. Given this contradiction and our inability to replicate the sequence result, the molecular species identification will be excluded from consideration in this study.

All confirmed sequences and those not compromised by damage are found on Genbank (Accessions OP547324-OP547329).

3. Traditional Fishing in the Tanana Valley

At least 15 species of native fish are used by modern indigenous communities in the Tanana River Valley, including both anadromous and freshwater forms (*48, 65-67, 69, 112*). Anadromous salmon are available in the lower and middle stretches of the river and include three species: *Oncorhynchus keta* (chum), *O. kisutch* (coho, silver), and *O. tshawytscha* (Chinook, king). Whitefish (subfamily Coregoninae) includes five species in three genera: *Coregonus pidschian* (humpback whitefish), *C. sardinella* (least cisco), *C. nasus* (broad whitefish), *Prosopium cylindraceum* (round whitefish), and *Stenodus leucichthys* (sheefish, inconnu). Whitefish are largely freshwater residents, although some individuals of some species may be anadromous in the lower Tanana (*113, 114*). A variety of other freshwater fish are harvested in the Tanana basin, including Arctic grayling (*Thymallus arcticus*), burbot (*Lota lota*), Northern pike (*Esox lucius*), longnose sucker (*Catostomus catostomus*), blackfish (*Dallia pectoralis*) and two species of char (*Salvelinus malma*, or Dolly Varden, and *S. namaycush*, or lake trout) (*48*). Note that char are also commonly referred to as "trout" but should not be confused with the trout species of *Oncorhynchus* (e.g., *O. mykiss*), which are not native to the Tanana Valley.

There is a natural dichotomy in the traditional fisheries of the Tanana basin: in the Upper Tanana, where salmon are absent, fishing focuses on whitefish, while in the Lower and Middle Tanana, fishing focuses on salmon, with whitefish of secondary importance (*65-69*). Throughout the Tanana Valley, the harvest of freshwater fish other than whitefish is fairly limited in most communities. Ethnographic reports suggest that these other freshwater fish were often "bycatch" during whitefish or salmon fishing or caught during indiscriminate mixed fish harvests (*65, 115*).

In an early ethnography for the region, McKennan (*68:62*) described fishing in Upper Tanana communities:

> Practically all fishing on the Upper Tanana is done in July. At this time the whitefish run from the lakes into the main river and are at their fattest. The streams draining the lakes are relatively small and slow moving, which greatly facilitates the use of fish weirs, and for a few weeks the natives congregate about the well-known fishing spots to take and dry their fish supply, which in addition to whitefish includes a few grayling, suckers, pike, and ling, or loche [burbot]. Virtually all their fish are taken by means of weirs in conjunction with either fish traps or dip nets. Weirs are constructed by driving posts into the bed of the stream. Branches are intertwined between these to form a fence, impenetrable except at the center where an opening is left. In this is placed a cylindrical-shaped trap with an opening like an inverted cone permitting easy entrance but difficult egress. . . . The hoop-shaped dip net, *uu*, is also used at the weir in place of the trap. The hoop is from 3 to 4 feet in diameter and the netting is of twined spruce roots or babiche. The fisherman stands on a platform built upon the weir.

Information on fish harvest patterns from more recent ethnographies and subsistence reports is summarized below for six fish taxa. The reports cover the Upper Tanana communities of Northway (*70*), Tetlin and Last Tetlin (*66, 67*), Tanacross (*116*), and Dot Lake (*71*); the Middle Tanana Salcha band (*69*); and the lower Tanana community of Minto (*65*).

3.1. Whitefish (subfamily Coregoninae)

*3.1.1. Harvest Season***.** In the Upper Tanana communities of, Northway, Tetlin, Last Tetlin, Tanacross, and Dot Lake, the whitefish harvest is concentrated in June or July, when whitefish migrate from lakes to rivers, although harvest may extend into the fall (*66-68, 70, 71, 112, 116*). Whitefish are never taken in deep winter, but may occasionally be harvested as late as December in Tanacross. For the middle Tanana Salcha band, Andrews (*69*) describes that whitefish are caught in the summer when whitefish descend from the lakes into streams. In the Lower Tanana community of Minto, whitefish are taken from May (after breakup) through October (prior to freeze up), with harvests concentrated in June and again in September/October (*65*). They are also "bycatch" in fishwheels and nets set for salmon in the summer. Occasionally whitefish are captured in nets set under the ice in late fall.

3.1.2. Technology. In Upper Tanana communities, whitefish are often procured using weirs in combination with dipnets or conical traps in streams and rivers and using set gillnets in rivers, streams, and lakes (*66, 67, 70, 71, 116*). Lt. Allen (*115*) observed the use of a dipnet to take white fish (as well other fish) in Last Tetlin ("Nandell's"). Traditionally, fish weirs were built of willow branches interwoven around posts set in the water (*67, 68*). McKennan (*68*) describes the use of a hoop-shaped dip net of 3-4 feet in diameter with netting constructed of entwined spruce roots, and Halpin (*66*) notes that the dip net used in Last Tetlin in the early 1980s was the same as described by McKennan but with netting made of cotton twine. Martin (*71:76*) points out that "nets placed in the river must be weighted at the bottom, while nets set in the slower creeks and lakes do not require weights". The Salcha people of the middle Tanana catch whitefish in a funnel-shaped basket at lake outlets (*69*), while in Minto, whitefish are captured using set gillnets placed in the river, as well as in fishwheels and in nets set for salmon.

3.1.3. Fishing Locations. In Northway, whitefish are harvested in small streams (by dip net and weir) and in eddies of larger rivers or in lakes where stream currents are flowing (using set

gillnets) (*70*). In Dot Lake, whitefish are harvested in the Tanana River and in tributary streams and lakes (by set gill net) (*71*). In the middle Tanana, whitefish are taken at lake outlets (*69*), while in Minto Andrews (*65*) describes that whitefish are captured in creeks, smaller rivers, sloughs, the Tanana River (using fishwheels), and lake outlets.

3.2. Salmon (*Oncorhynchus* spp.)

3.2.1. Harvest Season. For Upper Tanana communities (Northway, Tetlin, Tanacross, Dot Lake) salmon are harvested in June and July in distant locales, mostly on the Copper River (*66, 70, 71, 116*). Middle Tanana Salcha people harvest chinook salmon mostly in June and July, with some harvest in August, while chum and silver salmon are caught from late August to November. In Minto, salmon harvest extends from July through September and into October (*65*).

3.2.2. Technology. Nearly all of the salmon harvest in Upper Tanana communities occurs outside of the Tanana Valley on the Copper River using fishwheels owned by relatives or friends (*66, 70, 71, 116*). Residents of Dot Lake also infrequently used a dip net to harvest salmon on the Copper River (*71*). The Salcha people catch Chinook salmon are caught "in special traps which were set across the clearwater Salcha River about one-half mile up from its mouth" (*69:61*). The traps are described as "8 feet long and 2 feet in diameter at the entrance narrowing at the opposite end and are made from willows bound with a string of twisted willow bark" (*69:62*). Chum salmon are also caught in this trap, and fall chum and silver salmon are caught in the Tanana River using dipnets of willows and spruce roots. Few residents ever adopted the fishwheel. Andrews (*65*) describes that historically Minto residents used dip nets in the Tanana for summer salmon fishing; the dip net is described as having an elliptical frame around 3 feet in diameter with a short handle (around 5 feet) and was used from a canoe in the Tanana near mid-stream sandbars. Another type of dipnet was circular with a diameter of 3 feet; it was also used from a canoe drifting downstream. Also historically, short set nets in eddies were used for taking some kinds of salmon. Fishwheels were introduced sometime in the early 20th century. During Andrews' (*65*) study period in Minto in the early 1980s, fishwheels were used for salmon fishing but other technology was also used, including short gill nets set at strategic locations. Gill nets (vs fishwheel) were used exclusively by some families and in some river locations. Set gill nets include 50-foot chum salmon nets and shorter 20-25-foot nets.

3.2.3. Fishing Locations. Upper Tanana. Salmon fishing takes place outside of the area on the Copper River (*66, 70, 71, 116*). For the Salcha people, Chinook salmon fishing occurs on the Salcha River about ½ mile up from its mouth, while fall dog and silver salmon are caught in the Tanana River (using dipnets). In Minto, salmon fishing takes place in the silt-laden waters of the Tanana and its sloughs, and fish camps are located along the Tanana.

3.3. Burbot (*Lota lota*)

3.3.1 Harvest Season. The burbot harvest season is more variable than for other fish. In the Upper Tanana communities of Tetlin, Last Tetlin and Tanacross, burbot fishing occurs through the ice in the fall (October-December) (*66, 67, 116*). In Dot Lake, however, burbot fishing is heaviest in summer (late July through early September) (*71*), and for the Upper Tanana region in general McKennan (*68*) notes that fishing in July procured some burbot. In Northway, burbot fishing appears to be nearly year-round, from August through May (*70*). For the middle Tanana Salcha band, Andrews (*69*) mentions that burbot are sometimes taken in April on the Salcha

River, and burbot are mentioned as one of several "other fish" that are important in spring and fall as well as summer. In Minto, burbot are taken in August and September and occasionally in nets set under the ice in late fall (*65*).

3.3.2. Technology. In Dot Lake, Martin (*71*) describes that small quantities of burbot are taken with hook and line but larger quantities of burbot (and other fish) are taken with set gillnets in the Tanana River and in tributary lakes and creeks. McKennan (*68*) also notes that some burbot are caught in dip nets and traps set for whitefish. In Tetlin and Tanacross, burbot are harvested through the ice with a hand-held hook and line (*66, 70*). Halpin (*66:51*) describes that while the modern hook is metal, elders described a hook "fashioned from a dry branched limb." In Last Tetlin, burbot are taken under ice by hook with line, although it is unclear whether it is handheld (*67*). In Northway, burbot are taken using a "set longline" just before freeze-up from riverbanks or soon after freeze-up (*70*). In Northway, residents occasionally use rod and reel for burbot fishing in May and June (*70*). For the Salcha band, burbot fishing technology includes hook and line, although whether this is set line or handheld is not specified (*69*). In Minto, set gillnets are used for burbot (*65*). The same set gillnets used to capture whitefish also capture burbot; burbot are not necessarily targeted for harvest but are harvested along with whitefish, pike, and sucker; that is, a single net results in a mixed harvest.

3.3.3. Fishing Locations. In Northway, burbot are taken by set longline from river banks before freeze-up and on the rivers when they are covered thinly with ice (*70*). In Tetlin, burbot are taken through the ice on larger rivers before the ice gets too thick (*66*). In Dot Lake, burbot are taken by set gill net in the Tanana and in tributary lakes and creeks and by hook and line in clearwater creeks (*71*). The Salcha people take burbot in the spring on the Salcha River [with hook and line?] (*69*). In Minto, burbot are caught in gill nets in rivers, sloughs, and lakes (*65*).

3.4. Northern Pike (*Esox lucius*)

3.4.1. Harvest Season. Pike are harvested at different times of the year in different Upper Tanana communities. In Northway and Tetlin, the harvest extends from May to November (*66, 70*), although Guédon (*67*) shows pike fishing confined to October and early November for Tetlin as well as Last Tetlin. In Tanacross and Dot Lake, there are two seasons for pike fishing, one in June / July and the other in September / October, with occasional harvests in August, November and December (*71, 116*). For the Salcha band, pike are grouped with "other fish" that are important in spring through fall for immediate consumption (*69*). In Minto, the pike harvest is concentrated in May (at spring camps) and late October through November, with occasional harvest in June through September (*65*).

3.4.2. Technology. In Northway, pike are taken with rod and reel after breakup in May or June and by hooking or trapping small pike in funnel-shaped mesh traps at the edges of streams in September and just after freeze up, and by set longline through thin ice in October and November (*70*). In Tetlin, pike are taken by rod and reel in the summer, but this is viewed as recreational; in October just before freeze up, small pike are taken in small sloughs, river and lake shores using a hook attached to a long wooden pole; in the fall pike are taken with larger hooked poles, dip nets, or traps (*66*). The traps, now made of chicken wire, are 2 feet wide and around 4-5 feet long are placed in a narrow slough or under ice after freeze up in later fall on larger rivers (*66*). Pike are also taken though the ice using a hand-held line and hook (*66*). An

earlier report on Tetlin describes that pike are taken by "hooks or little nets in some lake outlets" and fish spears were "sometimes used" along the river as well as nets for ice fishing (*67:33*). Guédon (*67*) describes that a "team" is required for pike ice fishing, with someone breaking and deepening the ice channel, another person watching the net/trap downstream, and a third person trying to keep the fish from returning to the lake. In Tanacross, pike are harvested with nets at Lake Mansfield (*116*). In Dot Lake, small quantities of pike are taken with hook and line, while large quantities are taken with set gill nets in the Tanana River and tributary lakes and creeks, although the focus of the nets is whitefish (*71*). The Salcha people take pike by hook and line (*69*). In the lower Tanana community of Minto, pike are taken with nets (set for salmon or whitefish with pike as substantial bycatch), fishwheels (intended for salmon), hook and line (through the ice), long-handled dip net at the site of a fish fence with trap or with another kind of dip net used by holding the dip net from the bank of a creek to intercept pike (*65*). Pike are also caught by hooking or jigging through the ice and by rod and reel. Sometimes nets are set under the ice for pike and other fish. In the early 1900s a two-pronged leister was used to spear fish through the ice (*65*).

3.4.3. Fishing Locations. In the Upper Tanana communities of Northway and Tetlin, pike are taken by rod and reel in clear rivers and streams and in lakes; by hooked long pole or traps on the edges of streams, small sloughs, or lakes, and under ice on larger rivers (*66, 70*). Guédon (*67*) describes ice fishing for pike at lake outlets. In Dot Lake, pike are taken in clearwater creeks, and, using set gill nets, in the Tanana River and tributary lakes and creeks (*71*). In Minto, pike are caught at the outlets of lakes.

3.5. Grayling (*Thymallus arcticus*)

3.5.1. Harvest Season. Grayling harvest is variable among Upper Tanana communities. While in Northway the harvest extends from May through November, in Tetlin it is concentrated in May and June with occasional September fishing, and in Tanacross and Dot Lake it is concentrated in April with some harvest in May through August (*66, 70, 71, 116*). For the Salcha people of the middle Tanana, Andrews (*69*) notes that grayling are important in April as the ice began to break up, but are also procured in summer and fall. In Minto, grayling were not procured during the study year (1984) (*65*).

3.5.2. Technology. In Northway, grayling are taken with rod and reel from May through the summer and by "jigging" and "set longline" through the ice in late October through November (*70*). In Tetlin grayling are caught with rod and reel in the summer, which is viewed as a recreational activity (*66*). In Tanacross, grayling are taken by rod and reel in the summer (*116*). In Dot Lake, grayling are taken with hook and line, and "sometimes" as bycatch in whitefish set gill nets (*71*). For the Salcha band, grayling are obtained with hook and line (*69*).

3.5.3. Fishing Locations. In Northway, grayling are caught through the ice on rivers and on clear creeks and small lakes by rod and reel (*70*). In Tetlin grayling were caught in clear rivers and streams by rod and reel. For the Salcha band, grayling are taken on the Salcha River (*69*).

3.6. Longnose Sucker (*Catostomus catostomus*)

3.6.1. Harvest Season. In most communities on the Upper Tanana, the sucker harvest falls between May and September, although in Dot Lake they are occasionally taken into December

(*66, 70, 71, 116*). In Northway and Tetlin, suckers are reportedly used for dog food (*66, 70*). In Minto, suckers are harvested from May through September and are mostly fed to the dogs (*65*).

3.6.2. Technology. In Northway, suckers are harvested in small amounts with rod and reel (*70*). In Tetlin, suckers are not deliberately sought but are taken in nets set for whitefish (*66*). In Tanacross, suckers are taken with nets (along with whitefish and pike) (*116*). In Dot Lake, suckers are taken in gill nets (along with whitefish, pike, and burbot) (*71*). In Minto, suckers are taken with nets (along with whitefish and other fish); a single net (set for salmon or whitefish) will result in a mixed harvest including suckers (*65*).

3. 7. Winter Fishing

McKennan (*68:63*) notes that "winter fishing by either net or hook and line is not part of the Tanana culture," and this lack of winter fishing is echoed in several more recent ethnographies and reports. Guédon (*67:33*) states that in the Upper Tanana region "winter fishing is totally prevented by the thickness of the ice sheet on the lakes and rivers of the area," and that fishing for pike on the ice using nets is practiced but "only until the end of November, when the ice is think enough to stand the weight of the fishermen but not so thick as to prevent the cutting of a hole." Likewise, Halpin (*66*) notes that in Tetlin fishing through the ice occurs on fall just after freeze up but that the season is short lived because the ice thickens rapidly. Martin (*71*) describes that in Dot Lake by mid-winter the ice layer is too thick for ice fishing, and Andrews (*65*) mentions that in Minto under ice fishing occurs before the ice is too thick. However, Case (*70*) shows that in Northway there is nearly year-round fishing for burbot, although the technique is not described in the text, and difficulties about ice thickness are not mentioned.

Supplemental Figures

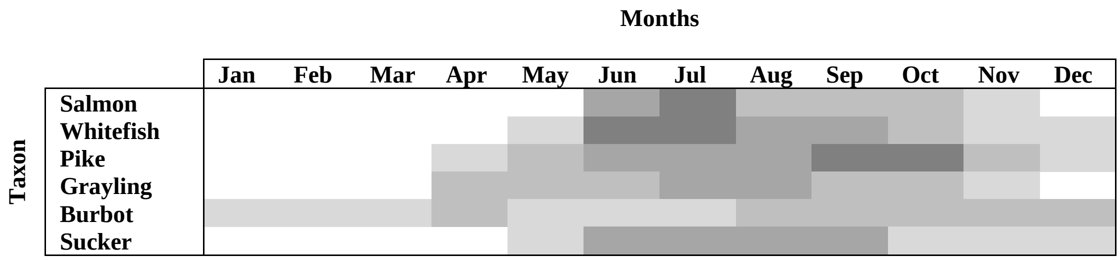

Fig. S1. Harvest season by fish taxon. Shading density corresponds to the number of communities (n=6) that harvest the species in that month and not to the number of fish taken.

Supplemental Tables

Table S1. Landscape setting and geomorphological characteristics of early fish-bearing sites in the Tanana Valley.

Site	Site Elevation (m asl)	Site Excavated Area (m2)	Sediment	Site landform	References
Broken Mammoth*	295	408	loess	bedrock bluff	(*16, 50, 117*);
Cook	310	6	loess	bedrock bluff	(*49, 97*)
Hollembaek	360	22	loess	bedrock knoll	this paper; (*45*)
Mead	360	287	loess	bedrock bluff	(*46, 50*)
Swan Point	320	89	loess	bedrock knoll	this paper; (*50*)
XBD-318	278	1	loess	alluvial terrace edge	(*41*).
Upward Sun River C3	325	240	loess	sand dune	(*32, 104*)

[*]Previously reported fish remains were not available for study.

Table S2. Radiocarbon ages for fish-bearing components.

**Site/Component**	**Age ^{14}C yr**	**Age cal BP**	**Dating basis**	**References**
Mead C2b (CZ4)	11,080 ± 20	13,090–12,930	component (mean pooled hearths age)	This study; (*23*)
Swan Point CZ3b	[*]	12,790–12,520[*]	modelled age range[*]	This study;. (*103*)
Cook CZ3	10,400 ± 60	12,600–12,000	component (single date)	(*15*)
Broken Mammoth CZ3[†]	10,290 ± 70	12,470–11,820	component (single hearth age)	(16, *50, 117*);
Mead C3a (CZ3b2)	10,270 ± 20	12,100–11,830	component (mean pooled hearths age)	This study
Upward Sun River C2[‡]	10,140 ± 40	11,940–11,410	component (mean pooled hearths age)	(*32*)
Swan Point CZ3a	[*]	11,890–11,430[*]	modelled age range[*]	This study; (*103*)
Upward Sun River C3	9990 ± 30	11,690–11,270	component (mean pooled hearths age)	(*32*)
XBD–318	10,830 ± 40	~12,000–11,300[§]	direct fish bone collagen date	(*18*)
Hollembaek	[*]	8800–7890[*]	modelled age range[*]	This study; (*118*)

Age cal BP is calibrated years before present (before 1950) expressed at 2 sigma and calculated with the IntCal20 terrestrial dataset.
[*]Modelled age ranges were calculated based on procedures outlines by Bronk Ramsey (*119*, *120*) and Hirasawa and Holmes (*103*). Modelled age ranges reflect calibrated (cal) BP modelled age beginning and end points for archaeological components or cultural zones based on a group of associated radiocarbon dates. These calibrated modelled age estimates do not have related uncalibrated radiocarbon mean pooled ages like those produced when a set of statistically similar uncalibrated radiocarbon dates are combined into a single average (pooled mean) age.
[†]Previously reported fish remains were not available for study.
[‡]Previously reported presence of salmon based on chemical profiling of hearth sediments (*19*).
[§]Age cal BP includes a correction for the marine reservoir effect (MRE); see *Supplementary Materials text* for details.

Table S3. Traditional subsistence fishes of the Tanana Valley.

Common name	Scientific name	Life history
Salmon	*Oncorhynchus sp.*	Anadromous
Whitefish	Subfamily Coregoninae	Resident migratory and anadromous
Arctic grayling	*Thymallus arcticus*	Resident migratory
Burbot	*Lota lota*	Resident migratory
Northern pike	*Esox lucius*	Resident migratory
Char	*Salvelinus sp.*	Resident migratory and resident non-migratory
Longnose sucker	*Catostomus catostomus*	Resident non-migratory
Blackfish	*Dallia pectoralis*	Resident non-migratory

Harvest intensity varies by taxon (*48*, *65*, *66*, *69-71*, *112*). Life history characterizations follow Seitz et al. (*121*).

Table S4. Diversity measures in fish assemblages.

Site	N Taxa	*D*	*H*	*E*	*R*
Mead C2b	1	1	0	0	1.431
Swan Point CZ3b	1	1	0	0	–
Cook CZ3	1	1	0	0	–
Mead C3a	2	0.886	0.229	0.33	–
XBD-318	1	1	0	0	–
Swan Point CZ3a	1	1	0	0	–
Upward Sun River C3	2	0.901	0.205	0.295	0.776
Hollembaek	4	0.398	1.05	0.757	2.861

N Taxa is the number of taxa. *D* is Simpson's diversity index (*122*), which expresses the probability that two randomly selected specimens will belong to the same taxon. *H* is the Shannon index (*123*), for which lower values indicate a less diverse assemblage and a value of zero indicates just one taxon. *E* is Evenness, which indicates how evenly the specimens in an assemblage are distributed among the different taxa; *E* varies from 0 to 1, where 1 indicates complete evenness. *R* is the richness index described by Odum et al. (*124*), which compensates for sample size differences by dividing the number of taxa by the natural log of the number of specimens collected.

Table S5. Fish element representation in the combined late Pleistocene/early Holocene assemblages.

Body Part	Element	N
**Identified Fish**		
Cranial	articular	7
	basibranchial	1
	basioccipital	2
	branchiostegal ray	38
	ceratohyal	5
	dentary	7
	epihyal	3
	hypohyal #2	1
	mandible	1
	maxilla	6
	mesethmoid	2
	opercle	2
	otolith	7
	parasphenoid	5
	premaxilla	2
	tooth	21
	quadrate	9
	Total Cranial	*113*
Pectoral Girdle	posttemporal	1
	supracleithrum	2
	Total Pectoral	*3*
Pelvic Girdle	interhaemal spine	1
	Total Pelvic	*1*
Vertebral Column	atlas vertebra	1
	caudal vertebra	93
	precaudal vertebra	56
	thoracic vertebra	39
	unspecified vertebra	291
	Total Vertebral	*480*
Unspecified Element	*Total Unspecified Element*	*30*
Total Identified Fish Specimens		***627***
**Unidentified Fish**		
Cranial	branchiostegal ray	1
	Total Cranial	*1*
Pelvic Gridle	interhaemal spine	1
	Total Pelvic	*1*
Vertebral Column	unspecified vertebra	145
	Total Vertebral	*145*
Unspecified Element	*Total Unspecified Element*	*336*
Total Unidentified Fish Specimens		***483***

Table S6. Body part representation in the Mead C3a burbot assemblage vs. the Upward Sun River C3 salmon assemblage.

		NISP	
**Body Part**	**Element**	**Mead C3a Burbot**	**Upward Sun River C3 Salmon**
**Element Specified**			
Cranial	articular	7	0
	basibranchial	0	1
	basioccipital	1	0
	branchiostegal ray	26	2
	ceratohyal	4	1
	dentary	0	6
	epihyal	3	0
	hypohyal #2	0	1
	maxilla	5	1
	mesethmoid	2	0
	opercle	1	1
	otolith	7	0
	parasphenoid	5	0
	premaxilla	2	0
	tooth	0	24
	quadrate	8	0
	Total Cranial	*71*	*37*
Pectoral Girdle	posttemporal	1	0
	supracleithrum	2	0
	Total Pectoral	*3*	*0*
Pelvic Girdle	interhaemal spine	0	1
	Total Pelvic	*0*	*1*
Vertebral Column	caudal vertebra	60	6
	precaudal vertebra	49	0
	thoracic vertebra	25	2
	unspecified vertebra	118	154
	Total Vertebral	*252*	*162*
Total Element Specified		***326***	***200***
Total Element Unspecified		***30***	***0***

Table S7. DNA species determinations for selected fish specimens.

ID	Site	Morphological ID	DNA ID	Replication?	Reference
Cultural					
3384	Mead C3a	*Lota lota* (burbot)	–	–	This study
3457	Mead C3a	*Lota lota* (burbot)	*Lota lota* (burbot)	No	This study
3724	Mead C3a	*Lota lota* (burbot)	*O. tshawytscha* (king salmon)*	No	This study
3732	Mead C3a	*Lota lota* (burbot)	–	–	This study
3745	Mead C3a	*Lota lota* (burbot)	–	–	This study
3836	Mead C3a	*Lota lota* (burbot)	–	–	This study
3997	Mead C3a	*Lota lota* (burbot)	–	–	This study
3428	Mead C3a	*Lota lota* (burbot)	–	–	This study
3519	Mead C3a	*Lota lota* (burbot)	–	–	This study
3438	Mead C3a	Coregoninae (whitefish)	–	–	This study
4137	Swan Point CZ3b	*Lota lota* (burbot)	*Lota lota* (burbot)	yes	This study
4181	Upward Sun River C3	*Oncorhynchus* sp. (salmon)	*O. keta* (chum salmon)	yes	(*17*)
4966	Upward Sun River C3	*Oncorhynchus* sp. (salmon)	*O. keta* (chum salmon)	yes	(*17*)
4850	XBD-318	*Oncorhynchus* sp. (salmon)	*O. keta* (chum salmon)	Yes	(*18*)
Mixed					
30	Lime Hills Cave S3	*Oncorhynchus* sp. (salmon)	*O. keta* (chum salmon)	yes	This study
31	Lime Hills Cave S3	*Oncorhynchus* sp. (salmon)	*O. keta* (chum salmon)	yes	This study
32	Lime Hills Cave S3	*Oncorhynchus* sp. (salmon)	*O. keta* (chum salmon)	yes	This study
33	Lime Hills Cave S3	*Oncorhynchus* sp. (salmon)	*O. keta* (chum salmon)	yes	This study
34	Lime Hills Cave S3	*Oncorhynchus* sp. (salmon)	*O. keta* (chum salmon)	yes	This study

*Incompatible with morphological determination.

Table S8. Bone collagen stable isotope values for selected fish specimens.

ID	Taxon	Site	Isotope Lab ID	$\delta^{13}C$ (‰)	$\delta^{15}N$ (‰)	%C	%N	Atomic C:N	Collagen Yield	Ref
3837	burbot	Mead C3a	UGAMS-47702	−21.2	–	–	–	–	–	This study
4137	burbot	Swan Point CZ3b	G-158428	−21.5	12.9	41.8	15.1	3.2	4.8	This study
4928	whitefish sp.	Hollembaek	G-166901	−23.1	8.4	39.8	14.2	3.3	13.6	(*18*)
4930	whitefish sp.	Hollembaek	G-166902	−25.1	10.2	41.7	15.6	3.1	13.1	(*18*)
4931	whitefish sp.	Hollembaek	G-166903	−24.8	10.2	41.8	15.5	3.1	10.6	(*18*)
4934	whitefish sp.	Hollembaek	G-166904	−24.7	10.3	42.1	15.6	3.2	12.2	(*18*)
4936	whitefish sp.	Hollembaek	G-166905	−25.3	10.2	42.1	15.7	3.1	14.5	(*18*)
4850	chum salmon	XBD-318	G-147956	−16.3	11.2	40.0	14.6	3.2	8.9	(*18*)
4966	chum salmon	Upward Sun River C3	G-118723	−15.1	12.6	43.1	15.8	3.2	6.9	(*17*)
30	chum salmon	Lime Hills Cave S3*	G-176216	−16.4	11.8	41.1	14.4	3.3	7.8	This study
31	chum salmon	Lime Hills Cave S3*	G-176220	−15.9	10.9	30.2	11.3	3.1	2.0	This study
32	chum salmon	Lime Hills Cave S3*	G-176217	−15.3	13.8	41.7	14.6	3.3	7.7	This study
33	chum salmon	Lime Hills Cave S3*	G-176218	−16.4	9.9	27.5	9.4	3.4	4.6	This study
34	chum salmon	Lime Hills Cave S3*	G176219	−16.7	11.0	32.6	12.0	3.2	2.2	This study

*Lime Hills Cave S3 is a mixed cultural/paleontological assemblage.

Dataset S1. Provenience and descriptive data for Alaskan fish remains analyzed in this study.

REFERENCES AND NOTES

1. M. D. Cannon, D. J. Meltzer, Early Paleoindian foraging: Examining the faunal evidence for large mammal specialization and regional variability in prey choice. *Quat. Sci. Rev.* **23**, 1955–1987 (2004).

2. T. A. Surovell, N. M. Waguespack, Human prey choice in the late Pleistocene and its relation to megafaunal extinctions, in *American Megafaunal Extinctions at the End of the Pleistocene*, G. Haynes, Ed. (Springer, 2009), pp. 77–105.

3. R. L. Lyman, *Vertebrate Taphonomy* (Cambridge Univ. Press, 1994).

4. R. C. Ferring, *The Archaeology and Paleoecology of the Aubrey Clovis Site (41DN479) Denton County, Texas* (Center for Environmental Archaeology, Department of Geography, University of North Texas, 2001).

5. B. Hockett, M. E. Adams, P. M. Lubinski, V. L. Butler, D. L. Jenkins, Late Pleistocene subsistence in the Great Basin: Younger Dryas-aged faunal remains from the Botanical Lens, Paisley Cave 2, Oregon. *J. Archaeol. Sci. Rep.* **13**, 565–576 (2017).

6. E. Johnson, F. C. Hill, The Lubbock Lake Landmark Late Pleistocene and Early Holocene paleoichthyofauna, Southern High Plains of Texas (USA). *Quat. Int.* **185**, 12–25 (2008).

7. R. Labarca, E. González-Guarda, Á. Lizama-Catalán, N. A. Villavicencio, J. Alarcón-Muñoz, F. Suazo-Lara, P. Oyanadel-Urbina, P. Soto-Huenchuman, C. Salazar, S. Soto-Acuña, K. E. Buldrini, Taguatagua 1: New insights into the late Pleistocene fauna, paleoenvironment, and human subsistence in a unique lacustrine context in central Chile. *Quat. Sci. Rev.* **238**, 106282 (2020).

8. J. M. Erlandson, M. L. Moss, Shellfish feeders, carrion eaters, and the archaeology of aquatic adaptations *Am. Antiq.* **66**, 413–432 (2001).

9. K. M. Stewart, D. Gifford-Gonzalez, An ethnoarchaeological contribution to identifying hominid fish processing sites. *J. Archaeol. Sci.* **21**, 237–248 (1994).

10. H. Russ, A. K. G. Jones, Fish remains in cave deposits; how did they get there?. *Cave Karst Sci.* **38**, 57–60 (2011).

11. J. A. M. Gingerich, T. R. Whyte, S. Whittaker. Misidentified Clovis-age fish bone at Shawnee-Minisink and the problem with single case studies in Late Pleistocene archaeology. *J. Archaeol. Sci. Rep.* **25**, 94–99 (2019).

12. L. N. Harris, E. B. Taylor, Pleistocene glaciations and contemporary genetic diversity in a Beringian fish, the broad whitefish, *Coregonus nasus* (Pallas): Inferences from microsatellite DNA variation. *J. Evol. Biol.* **23**, 72–86 (2010).

13. A. B. Shafer, C. I. Cullingham, S. D. Côté, D. W. Coltman, Of glaciers and refugia: A decade of study sheds new light on the phylogeography of northwestern North America. *Mol. Ecol.* **19**, 4589–4621 (2010).

14. M. A. Campbell, N. Takebayashi, J. A. López, Beringian sub-refugia revealed in blackfish (*Dallia*): Implications for understanding the effects of Pleistocene glaciations on Beringian taxa and other Arctic aquatic fauna. *BMC Evol. Biol.* **15**, 144 (2015).

15. F. B. Lanoë, J. D. Reuther, C. E. Holmes, G. W. L. Hodgins, Human paleoecological integration in subarctic eastern Beringia. *Quat. Sci. Rev.* **175**, 85–96 (2017).

16. D. R. Yesner, Human dispersal into interior Alaska: Antecedent conditions, mode of colonization, and adaptations. *Quat. Sci. Rev.* **20**, 315–327 (2001).

17. C. M. Halffman, B. A. Potter, H. J. McKinney, B. P. Finney, A. T. Rodrigues, D. Y. Yang, B. M. Kemp, Early human use of anadromous salmon in North America at 11,500 y ago. *Proc. Natl. Acad. Sci. U.S.A.* **112**, 12344–12348 (2015).

18. C. M. Halffman, B. A. Potter, H. J. McKinney, T. Tsutaya, B. P. Finney, B. M. Kemp, E. J. Bartelink, M. J. Wooller, M. Buckley, C. T. Clark, J. J. Johnson, B. L. Bingham, F. B. Lanoë, R. A. Sattler, J. D. Reuther, Ancient Beringian paleodiets revealed through multiproxy stable isotope analyses. *Sci. Adv.* **6**, eabc1968 (2020).

19. K. Choy, B. A. Potter, H. J. McKinney, J. D. Reuther, S. W. Wang, M. J. Wooller, Chemical profiling of ancient hearths reveals recurrent salmon use in Ice Age Beringia. *Proc. Natl. Acad. Sci. U.S.A.* **113**, 9757–9762 (2016).

20. N. A. Endacott, thesis, Washington State University, Pullman, WA (2008).

21. H. Larsen, *Trail Creek: Final Report on the Excavation of Two Caves on Seward Peninsula, Alaska* (Ejnar Munksgaard, 1968).

22. C. R. Harington, J. Cinq-Mars, *Bluefish Caves—Fauna and Context* (Beringian Research Notes, 2008).

23. B. A. Potter, C. E. Holmes, D. R. Yesner, Technology and economy among the earliest prehistoric foragers in interior eastern Beringia, in *Paleoamerican Odyssey*, K. E. Graf, C. V. Ketron, M. R. Waters, Eds. (Center for the Study of the First Americans, 2013), pp. 81–103.

24. B. T. Wygal, K. E. Krasinski, C. E. Holmes, B. A. Crass, Holzman South: A late Pleistocene archaeological site along Shaw Creek, Tanana Valley, Interior Alaska. *PaleoAmerica* **4**, 90–93 (2018).

25. B. A. Potter, J. D. Reuther, "Site chronology" in *Archaeological investigations at Delta River Overlook*, B. A. Potter, Ed. (Archaeology GIS Laboratory, University of Alaska Fairbanks, 2018), pp. 58–70.

26. B. A. Potter, J. F. Baichtal, A. B. Beaudoin, L. Fehren-Schmitz, C. V. Haynes, V. T. Holliday, C. E. Holmes, J. W. Ives, R. L. Kelly, B. Llamas, R. S. Malhi, D. S. Miller, D. Reich, J. D. Reuther, S. Schiffels, T. A. Surovell, Current evidence allows multiple models for the peopling of the Americas. *Sci. Adv.* **4**, eaat5473 (2018).

27. T. Goebel, M. R. Waters, D. H. O'Rourke. The late Pleistocene dispersal of modern humans in the Americas. *Science* **319**, 1497–1502 (2008).

28. D. J. Meltzer, *First Peoples in a New World: Populating Ice Age America* (Cambridge Univ. Press, 2021)

29. N. Endacott, R. Ackerman, Bird and fish remains from Lime Hills Cave, SW Alaska. *Curr. Res. Pleist.* **26**, 51–53 (2009).

30. K. M. Bovy, M. A. Etnier, V. L. Butler, S. K. Campbell, J. D. Shaw, Using bone fragmentation records to investigate coastal human ecodynamics: A case study from Čḯxwicən (Washington State, USA). *J. Archaeol. Sci. Rep.* **23**, 1168–1186 (2019).

31. R. J. Piorkowski, thesis, University of Alaska Fairbanks, Fairbanks, AK (1995).

32. B. A. Potter, J. D. Irish, J. D. Reuther, H. J. McKinney, New insights into Eastern Beringian mortuary behavior: A terminal Pleistocene double infant burial at Upward Sun River. *Proc. Natl. Acad. Sci. U.S.A.* **111**, 17060–17065 (2014).

33. A. M. Rick, Bird medullary bone: A seasonal dating technique for faunal analysts, in *Bulletin (Canadian Archaeological Association)* (Canadian Archaeological Association, 1975), pp. 183–190.

34. R. J. Ritchie, S. Ambrose, Distribution and population status of bald eagles (*Haliaeetus leucocephalus*) in interior Alaska. *Arctic* **49**, 120–128 (1996).

35. B. A. Potter, Exploratory models of intersite variability in mid to late Holocene central Alaska. *Arctic* **61**, 407–425 (2008).

36. B. A. Potter, thesis, University of Alaska Fairbanks, Fairbanks, AK (2005).

37. B. A. Potter, Late Pleistocene and early Holocene assemblage variability in central Alaska, in *From the Yenisei to the Yukon: Interpreting Lithic Assemblage Variability in Late Pleistocene/early Holocene Beringia*, T. E. Goebel, I. Buvit, N. Easton, R. Ackerman, J. Baus, Eds. (Texas A&M Press, 2011), pp. 215–233.

38. C. E. Holmes, Broken mammoth, in *American Beginnings: The Prehistory and Paleoecology of Beringia*, F. H. West, Ed. (Univ. of Chicago Press, 1996), pp. 312–318.

39. F. B. Lanoë, J. D. Reuther, C. E. Holmes, Task-specific sites and Paleoindian landscape use in the Shaw Creek Flats, Alaska. *J. Archaeol. Method Theory* **25**, 818–838 (2018).

40. C. E. Holmes, R. VanderHoek, T. E. Dilley, Swan Point, in *American Beginnings: The Prehistory and Paleoecology of Beringia*, F. H. West, Ed. (Univ. of Chicago Press, 1996), pp. 319–322.

41. B. A. Potter, E. P. Gaines, P. M. Bowers, M. Proue, “Results of the 2006 cultural resource survey of proposed Alaska railroad northern rail extension routes and ancillary facilities, Alaska” (Technical Report #278b-c, Northern Land Use Research Inc., 2007).

42. C. E. Holmes, Tanana River Valley archaeology circa 14,000 to 9000 B.P. *Arct. Anthropol.* **38**, 154–170 (2001).

43. C. E. Holmes, The Beringian and transitional periods in Alaska: Technology of the East Beringian tradition as viewed from Swan Point, in *From the Yenisei to the Yukon: Interpreting Lithic Assemblage Variability in Late Pleistocene/Early Holocene Beringia*, T. E. Goebel, I. Buvit, Eds. (Center for the Study of the First Americans, Texas A&M Univ. Press, 2011), pp. 179–191.

44. F. B. Lanoë, J. D. Reuther, C. R. Holloway, C. E. Holmes, J. R. Kielhofer, The Keystone Dune Site: A Bølling-Allerød hunting camp in Eastern Beringia. *PaleoAmerica* **4**, 151–161 (2018).

45. F. B. Lanoë, J. D. Reuther, C. E. Holmes, B. A. Potter, Small mammals and paleovironmental context of the terminal Pleistocene and early Holocene human occupation of central Alaska. *Geoarchaeology* **35**, 164–176 (2020).

46. B. A. Potter, P. J. Gilbert, C. E. Holmes, B. A. Crass, The Mead Site: A Late Pleistocene-Holocene stratified site in central Alaska. *Curr. Res. Pleist.* **28**, 73–75 (2011).

47. J. D. Reuther, B. A. Potter, C. E. Holmes, J. K. Feathers, F. B. Lanoë, J. Kielhofer, The Rosa-Keystone Dunes Field: The geoarchaeology and paleoecology of a late Quaternary stabilized dune field in Eastern Beringia. *Holocene* **26**, 1939–1953 (2016).

48. Alaska Department of Fish and Game, Community Subsistence Information System (Alaska Department of Fish and Game, 2022); www.subsistence.adfg.state.ak.us.

49. J. D. Reuther, thesis, University of Arizona, Tucson, AZ (2013).

50. T. E. Dilley, thesis, University of Arizona, Tucson, AZ (1998).

51. J. K. J. Van Houdt, L. De Cleyn, A. Perretti, F. A. M. Volckaert, A mitogenic view on the evolutionary history of the Holarctic freshwater gadoid, burbot *(Lota lota*). *Mol. Ecol.* **14**, 2445–2457 (2005).

52. A. Skog, L. A. Vøllestad, N. C. Stenseth, A. Kasumyan, K. S. Jakobsen, Circumpolar phylogeography of the northern pike (*Esox lucius*) and its relationship to the Amur pike (*E. reichertii*). *Front. Zool.* **11**, 67 (2014).

53. B. E. Caissie, J. Brigham-Grette, K. T. Lawrence, T. D. Herbert, M. S. Cook, Last Glacial Maximum to Holocene sea surface conditions at Umnak Plateau, Bering Sea, as inferred from diatom, alkenone, and stable isotope records. *Paleoceanography* **25**, PA1206 (2010).

54. Z. R. Van Orsdel, thesis, Idaho State University, Pocatello, ID (2019).

55. M. B. Abbott, B. P. Finney, M. E. Edwards, K. R. Kelts, Lake-Level reconstruction and paleohydrology of Birch Lake, Central Alaska, based on seismic reflection profiles and core transects. *Quatern. Res.* **53**, 154–166 (2000).

56. V. A. Barber, B. P. Finney, Late quaternary paleoclimatic reconstructions for interior Alaska based on paleolake-level data and hydrologic models. *J. Paleolimnol.* **24**, 29–41 (2000).

57. M. S. Finkenbinder, M. B. Abbott, M. E. Edwards, C. T. Langdon, B. A. Steinman, B. P. Finney, A 31,000 year record of paleoenvironmental and lake-level change from Harding Lake, Alaska, USA. *Quat. Sci. Rev.* **87**, 98–113 (2014).

58. T. A. Ager, Holocene vegetational history of Alaska, in *Late Quaternary Environments of the United States, v. 2, The Holocene,* H. E. Wright, Jr., Ed. (Univ. of Minnesota Press, 1983), pp. 128–141.

59. N. H. Bigelow, J. D. Reuther, K. L. Wallace, É. Saulnier-Talbot, K. Mulliken, M. J. Wooller, Late-glacial paleoecology of the middle Susitna Valley, Alaska: Environmental Context for Human Dispersal. *Front. Earth Sci.* **7**, 43 (2019).

60. M. J. Wooller, J. Kurek, B. V. Gaglioti, L. C. Cwynar, N. Bigelow, J. D. Reuther, C. Gelvin-Reymiller, J. P. Smol, An 11,200 year paleolimnological perspective for emerging archaeological findings at Quartz Lake, Alaska. *J. Paleolimnol.* **48**, 83–99 (2012).

61. T. A. Ager, L. B. Brubaker, Quaternary palynology and vegetational history of Alaska, in *Pollen Records of Late-Quaternary North American Sediments*, V. M. Bryant, Jr., R. G. Holloway, Eds. (American Association of Stratigraphic Palynologists Foundation, 1985), pp. 353–384.

62. N. H. Bigelow, M. E. Edwards, A 14,000yr paleoenvironmental record from Windmill Lake, Central Alaska: Lateglacial and Holocene vegetation in the Alaska range. *Quat. Sci. Rev.* **20**, 203–215 (2001).

63. J. R. Kielhofer, J. E. Tierney, J. D. Reuther, B. A. Potter, C. E. Holmes, F. B. Lanoë, J. A. Esdale, M. J. Wooller, N. H. Bigelow. BrGDGT temperature reconstruction from interior Alaska: Assessing 14,000 years of deglacial to Holocene temperature variability and potential effects on early human settlement. *Quat. Sci. Rev.* **303**, 107979 (2023).

64. B. A. Potter, A first approximation of Holocene inter-assemblage variability in central Alaska. *Arct. Anthropol.* **45**, 89–113 (2008).

65. E. F. Andrews, "The harvest of fish and wildlife for subsistence by residents of Minto, Alaska" (Technical Report 137, Alaska Department of Fish and Game, 1988).

66. L. Halpin, "Living off the land: Contemporary subsistence in Tetlin, Alaska" (Technical Report 149,Alaska Department of Fish and Game, 1987).

67. M.-F. Guédon, *People of Tetlin, Why Are You Singing?* (National Museums of Canada, 1974).

68. R. A. McKennan, *The Upper Tanana Indians* (Yale University, 1959).

69. E. F. Andrews, thesis, University of Alaska Fairbanks, Fairbanks, AK (975).

70. M. F. Case, "Wild resource use in Northway, Alaska" (Technical Report 132, Alaska Department of Fish and Game, 1986).

71. G. Martin, “Use of natural resources by the residents of Dot Lake, Alaska” (Technical Report 78, Alaska Department of Fish and Game, 1983).

72. B. A. Potter, Models of faunal processing and economy in early Holocene interior Alaska. *Environ. Archaeol.* **12**, 3–23 (2007).

73. A. Heppner, thesis, University of Alaska Anchorage, Anchorage, AK (2017).

74. J. F. Hoffecker, Innovation and technological knowledge in the Upper Paleolithic of Northern Eurasia. *Evol. Anthropol.* **14**, 186–198 (2005).

75. D. R. Yesner, Faunal extinction, hunter-gatherer foraging strategies, and subsistence diversity among eastern Beringian Paleoindians, in *Foragers of the Terminal Pleistocene in North America*, R. B. Walker, B. N. Driskell, Eds. (Univ. of Nebraska Press, 2007), pp. 15–31.

76. F. B. Lanoë, C. E. Holmes, Animals as raw material in Beringia: Insights from the site of Swan Point CZ4B, Alaska. *Am. Antiq.* **81**, 682–696 (2016).

77. R. L. Kelly, L. C. Todd, Coming into the country: Early Paleoindian hunting and mobility. *Am. Antiq.* **53**, 231–244 (1988).

78. L. R. Binford, Willow smoke and dogs' tails: Hunter-gatherer settlement systems and archaeological site formation. *Am. Antiq.* **45**, 4–20 (1980).

79. J. M. Erlandson, T. C. Rick, T. J. Braje, M. Casperson, B. Culleton, B. Fulfrost, T. Garcia, D. A. Guthrie, N. Jew, D. J. Kennett, M. L. Moss, L. Reeder, C. Skinner, J. Watts, L. Willis, Paleoindian seafaring, maritime technologies, and coastal foraging on California’s Channel Islands. *Science* **331**, 1181–1185 (2011).

80. R. L. Lyman, Quantitative units and terminology in zooarchaeology. *Am. Antiq.* **59**, 36–71 (1994).

81. I. McKechnie, Zooarchaeological analysis of the Indigenous fishery at the Huu7ii Big House and back terrace, Huu-ay-aht territory, southwestern Vancouver Island, in *Huu7ii: Household*

Archaeology at a Nuu-chah-nulth Village Site in Barkley Sound, A. D. McMillan, D. E. St. Claire, Eds. (Archaeology Press, 2012), pp. 154–186.

82. P. Szpak, J. Z. Metcalfe, R. A. Macdonald, Best practices for calibrating and reporting stable isotope measurements in archaeology. *J. Archaeol. Sci. Rep.* **13**, 609–616 (2017).

83. J. L. Barta, C. Monroe, B. M. Kemp, Further evaluation of the efficacy of contamination removal from bone surfaces. *Forensic Sci. Int.* **231**, 340–348 (2013).

84. B. M. Kemp, C. Monroe, K. G. Judd, E. Reams, C. Grier, Evaluation of methods that subdue the effects of polymerase chain reaction inhibitors in the study of ancient and degraded DNA. *J. Archaeol. Sci.* **42**, 373–380 (2014).

85. B. M. Kemp, R. S. Malhi, J. McDonough, D. A. Bolnick, J. A. Eshleman, O. Rickards, C. Martinez-Labarga, J. R. Johnson, J. G. Lorenz, E. J. Dixon, T. E. Fifield, T. H. Heaton, R. Worl, D. G. Smith, Genetic analysis of early holocene skeletal remains from Alaska and its implications for the settlement of the Americas. *Am. J. Phys. Anthropol.* **132**, 605–621 (2007).

86. M. L. Moss, K. G. Judd, B. M. Kemp, Can salmonids (*Oncorhynchus* spp.) be identified to species using vertebral morphometrics? A test using ancient DNA from Coffman Cove, Alaska. *J. Archaeol. Sci.* **41**, 879–889 (2014).

87. C. Hänni, T. Brousseau, V. Laudet, D. Stehelin, Isopropanol precipitation removes PCR inhibitors from ancient bone extracts. *Nucleic Acids Res.* **23**, 881–882 (1995).

88. B. J. Wilson, S. J. Crockford, J. W. Johnson, R. S. Malhi, B. M. Kemp, Genetic and archaeological evidence for a former breeding population of Aleutian Cackling Goose (Branta hutchinsii leucopareia) on Adak Island, central Aleutians, Alaska. *Can. J. Zool.* **89**, 732–743 (2011).

89. L. G. Jordan, C. A. Steele, G. H. Thorgaard, Universal mtDNA primers for species identification of degraded bony fish samples. *Mol. Ecol. Resour.* **10**, 225–228 (2010).

90. E. Palmer, S. Tushingham, B. M. Kemp, Human use of small forage fish: Improved ancient DNA species identification techniques reveal long term record of sustainable mass harvesting of smelt fishery in the northeast Pacific Rim. *J. Archaeol. Sci.* **99**, 143–152 (2018).

91. B. M. Johnson, B. M. Kemp, Rescue PCR: Reagent-rich PCR recipe improves amplification of degraded DNA extracts. *J. Archaeol. Sci. Rep.* **11**, 683–694 (2017).

92. B. M. Kemp, K. Judd, C. Monroe, J. W. Eerkens, L. Hilldorfer, C. Cordray, R. Schad, E. Reams, S. G. Ortman, T. A. Kohler, Prehistoric mitochondrial DNA of domesticate animals supports a 13th century exodus from the northern US southwest. *PLOS ONE* **12**, e0178882 (2017).

93. B. M. Kemp, B. Bingham, R. Frome, M. Labonte, E. Palmer, E. S. Parsons, K. W. Gobalet, J. Rosenthal, Subduing the influence of PCR inhibitors on amplifying aged, degraded, and low copy number DNA: PCR enhancer cocktail-p and rescue PCR. *PLOS ONE* **15**, e0234745 (2020).

94. R. B. Alley, GISP2 Ice Core Temperature and Accumulation Data. IGBP Pages/World Data Center for Paleoeclimatology Data Contribution Series #2004-013 (NOAA/NGDC Paleoclimatology Program, 2004).

95. B. A. Potter, Radiocarbon chronology of central Alaska: Technological continuity and economic change. *Radiocarbon* **50**, 181–204 (2008).

96. J. Kielhofer, C. Miller, J. Reuther, C. Holmes, B. Potter, F. Lanoë, J. Esdale, B. Crass, The micromorphology of loess-paleosol sequences in central Alaska: A new perspective on soil formation and landscape evolution since the Late Glacial period (c. 16,000 cal yr BP to present). *Geoarchaeology* **35**, 701–728 (2020).

97. L. S. Timmons, G. A. Pearse, "Abundance of the northern pike populations of George, Volmar, and T lakes with estimates of age, sex, and length composition, 1988" (Alaska Department of Fish and Game, 1989).

98. P. J. Gilbert, thesis, University of Alaska Fairbanks, Fairbanks, AK (2011).

99. A. A. Little, thesis, University of Alaska Fairbanks, Fairbanks, AK (2013).

100. C. E. Holmes, New data pertaining to Swan Point, the oldest microblade site known in Alaska. *Curr. Res. Pleist.* **15**, 21–22 (1998).

101. C. E. Holmes, The Taiga period: Holocene archaeology of the northern boreal forest, Alaska. *Alaska J. Anthropol.* **6**, 69–81 (2008).

102. Y. A. Gómez Coutouly, C. E. Holmes, The microblade industry from Swan Point Cultural Zone 4b: Technological and cultural implications from the earliest human occupation in Alaska. *Am. Antiq.* **83**, 735–752 (2018).

103. Y. Hirasawa, C. E. Holmes, The relationship between microblade morphology and production technology in Alaska from the perspective of the Swan Point site. *Quat. Int.* **442**, 104–117 (2017).

104. B. A. Potter, J. D. Irish, J. D. Reuther, C. Gelvin-Reymiller, V. T. Holliday, A terminal Pleistocene child cremation and residential structure from eastern Beringia. *Science* **331**, 1058–1062 (2011).

105. J. Southon, D. Fedje, A post-glacial record of ^{14}C reservoir ages for the British Columbia Coast. *Canadian J. Archaeol.* **27**, 95–111 (2003).

106. A. de Flamingh, E. K. Mallott, A. L. Roca, A. S. Boraas, R. S. Malhi, Species identification and mitochondrial genomes of ancient fish bones from the Riverine Kachemak tradition of the Kenai Peninsula, Alaska. *Mitochondrial DNA Part B* **3**, 409–411 (2018).

107. N. Schmuck, J. Reuther, J. F. Baichtal, R. J. Carlson, Quantifying marine reservoir effect variability along the Northwest Coast of North America. *Quatern. Res.* **103**, 160–181 (2021).

108. P. M. Ledger, V. Forbes, E. Masson-MacLean, R. A. Knecht, Dating and digging stratified archaeology in circumpolar North America: A view from Nunalleq, southwestern Alaska. *Arctic* **69**, 378–458 (2016).

109. N. Zhang, N. Song, T. Gao, The complete mitochondrial genome of *Lota lota* (Gadiformes: Gadidae). *Mitochondrial DNA A DNA Mapp. Seq. Anal.* **27**, 1437–1438 (2016).

110. K. H. Brown, R. E. Drew, L. A. Weber, G. H. Thorgaard, Intraspecific variation in the rainbow trout mitochondrial DNA genome. *Comp. Biochem. Physiol. Part D Genomics Proteomics* **1**, 219–226 (2006).

111. C. Grier, K. Flanigan, M. Winters, L. G. Jordan, S. Lukowski, B. M. Kemp, Using ancient DNA identification and osteometric measures of archaeological Pacific Salmon vertebrae for reconstructing salmon fisheries and site seasonality at Dionisio Point, British Columbia. *J. Archaeol. Sci.* **40**, 544–555 (2013).

112. J. R. Marcotte, "Wild fish and game harvest an use by residents of five Upper Tanana communities, Alaska, 1987–88" (Technical Report 168, Alaska Department of Fish and Game, 1991).

113. R. J. Brown, C. Brown, N. M. Braem, W. K. I. Carter, N. Legere, L. Slayton, "Whitefish biology, distribution, and fisheries in the Yukon and Kuskokwim River drainages in Alaska: A synthesis of available information" (US Fish and Wildlife Service, Alaska Fisheries Data Series Number 2012-4, 2012).

114. R. J. Brown, N. Bickford, K. Severin, Otolith trace element chemistry as an indicator of anadromy in Yukon River drainage coregonine fishes. *Trans. Am. Fish. Soc.* **136**, 678–690 (2007).

115. H. T. Allen, *Report of an Expedition to the Copper, Tananá, and Kóyukuk Rivers, in the Territory of Alaska, in the Year 1885* (Government Printing Office, 1887).

116. T. L. Haynes, M. Case, J. A. Fall, L. Halpin, M. Robert, "The use of Copper River salmon and other wild resources by Upper Tanana communities" (Technical Report 115, Alaska Department of Fish and Game, 1984).

117. K. Krasinski, D. R. Yesner, Late Pleistocene/early Holocene site structure in Beringia: a case study from the Broken Mammoth site, interior Alaska. *Alaska J. Anthropol.* **6**, 27–41 (2008).

118. F. B. Lanoë, J. Reuther, Environmental change and human ecology in central Alaska during the early Holocene: Hollembaek's Hill, paper presented at the 83rd Annual Meeting for the Society for American Archaeology, Albuquerque, NM, 10 to 14 April 2019.

119. C. Bronk Ramsey, Bayesian analysis of radiocarbon dates. *Radiocarbon* **51**, 337–360 (2009).

120. C. Bronk Ramsey, Dealing with outliers and offsets in radiocarbon dating. *Radiocarbon* **51**, 1023–1045 (2009).

121. A. C. Seitz, K. Moerlein, M. D. Evans, A. E. Rosenberger, Ecology of fishes in a high-latitude, turbid river with implications for the impacts of hydrokinetic devices. *Rev. Fish Biol. Fisheries* **21**, 481–496 (2011).

122. E. H. Simpson, Measurement of diversity. *Nature* **163**, 688–688 (1949).

123. C. E. Shannon, A mathematical theory of communication. *Bell Syst. Tech. J.* **27**, 379–423 (1948).

124. H. T. Odum, J. E. Cantlon, L. S. Kornicker, An organizational hierarchy postulate for the interpretation of species-individual distributions, species entropy, ecosystem evolution, and the meaning of a species-variety index. *Ecology* **41**, 395–395 (1960).
